# Design, synthesis, *in vitro* antiproliferative activity and apoptosis-inducing studies of 1-(3′,4′,5′-trimethoxyphenyl)-3-(2′-alkoxycarbonylindolyl)-2-propen-1-one derivatives obtained by a molecular hybridisation approach

**DOI:** 10.1080/14756366.2018.1493473

**Published:** 2018-08-24

**Authors:** Delia Preti, Romeo Romagnoli, Riccardo Rondanin, Barbara Cacciari, Ernest Hamel, Jan Balzarini, Sandra Liekens, Dominique Schols, Francisco Estévez-Sarmiento, José Quintana, Francisco Estévez

**Affiliations:** a Department of Chemical and Pharmaceutical Sciences, University of Ferrara, Ferrara, Italy;; b Screening Technologies Branch, Developmental Therapeutics Program, Division of Cancer Treatment and Diagnosis, Frederick National Laboratory for Cancer Research, National Cancer Institute, National Institutes of Health, Frederick, MD, USA;; c Rega Institute for Medical Research, KU Leuven, Laboratory of Virology and Chemotherapy, Leuven, Belgium;; d Department of Biochemistry and Molecular Biology, Research Institute in Biomedical and Health Sciences (IUIBS), University of Las Palmas de Gran Canaria (ULPGC), Spain

**Keywords:** Microtubule, apoptosis, tumour cell growth, indole derivatives, structure–activity relationship

## Abstract

Inhibition of microtubule function using tubulin targeting agents has received growing attention in the last several decades. The indole scaffold has been recognized as an important scaffold in the design of novel compounds acting as antimitotic agents. Indole-based chalcones, in which one of the aryl rings was replaced by an indole, have been explored in the last few years for their anticancer potential in different cancer cell lines. Eighteen novel (3′,4′,5′-trimethoxyphenyl)-indolyl-propenone derivatives with general structure **9** were synthesized and evaluated for their antiproliferative activity against a panel of four different human cancer cell lines. The highest IC_50_ values were obtained against the human promyelocytic leukemia HL-60 cell line. This series of chalcone derivatives was characterized by the presence of a 2-alkoxycarbonyl indole ring as the second aryl system attached at the carbonyl of the 3-position of the 1-(3′,4′,5′-trimethoxyphenyl)-2-propen-1-one framework. The structure–activity relationship (SAR) of the indole-based chalcone derivatives was investigated by varying the position of the methoxy group, by the introduction of different substituents (hydrogen, methyl, ethyl or benzyl) at the *N*-1 position and by the activity differences between methoxycarbonyl and ethoxycarbonyl moieties at the 2-position of the indole nucleus. The antiproliferative activity data of the novel synthesized compounds revealed that generally *N*-substituted indole analogues exhibited considerably reduced potency as compared with their parent *N*-unsubstituted counterparts, demonstrating that the presence of a hydrogen on the indole nitrogen plays a decisive role in increasing antiproliferative activity. The results also revealed that the position of the methoxy group on the indole ring is a critical determinant of biological activity. Among the synthesized derivatives, compound **9e**, containing the 2-methoxycarbonyl-6-methoxy-*N*-1*H*-indole moiety exhibited the highest antiproliferative activity, with IC_50_ values of 0.37, 0.16 and 0.17 μM against HeLa, HT29 and MCF-7 cancer cell lines, respectively, and with considerably lower activity against HL-60 cells (IC_50_: 18 μM). This derivative also displayed cytotoxic properties (IC_50_ values ∼1 μM) in the human myeloid leukemia U-937 cell line overexpressing human Bcl-2 (U-937/Bcl-2) via cell cycle progression arrest at the G_2_-M phase and induction of apoptosis. The results obtained also demonstrated that the antiproliferative activity of this molecule is related to inhibition of tubulin polymerisation. The presence of a methoxy group at the C5- or C6-position of the indole nucleus, as well as the absence of substituents at the *N*-1-indole position, contributed to the optimal activity of the indole-propenone-3′,4′,5′-trimethoxyphenyl scaffold.

## Introduction

The microtubule system of eukaryotic cells, a dynamic polymeric protein machinery composed of α- and β-tubulin heterodimers, is a critical element in a variety of cellular functions, including determination and maintenance of cell shape, organisation of intracellular architecture, secretion, chromosome segregation during mitosis, cellular transport, regulation of motility and organelle transport inside the cell^1^^,^^2^. By interfering with microtubule dynamics, inhibiting or enhancing the polymerisation of tubulin into microtubules, tumour cells become arrested in mitosis, followed by cell death by apoptosis or necrosis^3^^,^^4^. Natural and synthetic small molecules that interfere with the assembly of tubulin into microtubules have antimitotic activity as a result of inhibition of tubulin polymerisation^5–7^. Inhibition of tubulin polymerisation can also disrupt the formation of tumour vasculature, making the microtubules a highly attractive target for the development of potential new chemotherapeutic agents^8^^,^^9^. Although many synthetic tubulin inhibitors have been synthesized, there is still a need to identify novel molecules that target microtubules^10–12^. Such compounds would be ideally characterized by a relatively simple structure and be easy to prepare in a cost-effective way^13^.

**Figure 1.**
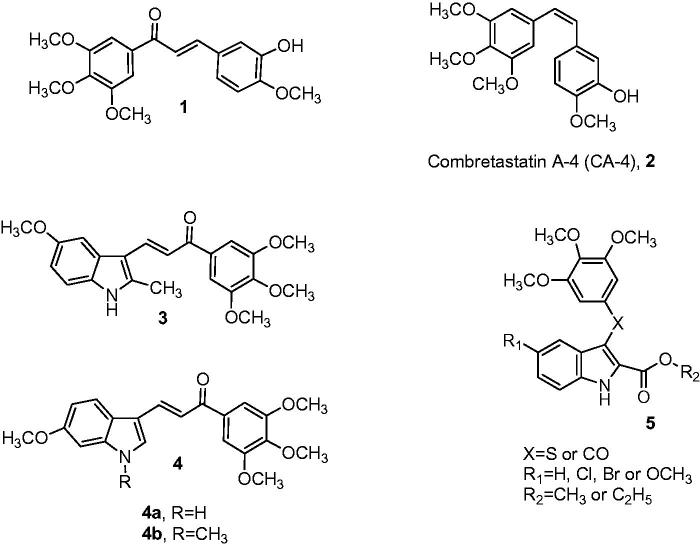
Structure of chalcone (**1**) reported by Ducki et al., combretastatin A-4 (CA-4, **2**), indole-based chalcone derivatives **3–4** and general structure (**5**) of methyl/ethyl 3-[(3′,4′,5′-trimethoxyphenyl)thio/carbonyl]-5-substituted-1*H*-indole-2-carboxylates reported by De Martino et al.

Chalcones (1,3-diphenyl-3-propen-1-ones) are antitumor agents characterized by the presence of two aromatic rings linked by a three-carbon α,β-unsaturated system (Figure 1). A large number of synthetic chalcones have been shown to have potent antiproliferative activity against cancer cell lines, exhibiting antimitotic properties caused by inhibition of tubulin assembly through binding to the colchicine site^14^^,^^15^. Considering structure–activity relationship studies, derivatives that contain a 3′,4′,5′-trimethoxyphenyl ring as one of the aryl rings is thought to be of great interest for anticancer activity^16^. From the wide number of synthetic chalcones tested for their cell growth inhibitory activity, Ducki et al.^18^ discovered compound **1**^17^ possessing the same aromatic substitution pattern as the naturally occurring stilbene derivative combretastatin A-4 (CA-4, **2**). This derivative showed antiproliferative effects against various cancer cell lines by acting as an antimitotic agent by the disruption of microtubule polymerisation.

In the past two decades, the indole molecular skeleton has been widely employed as a versatile pharmacophore for the development of an increasing number of chemically diverse small molecules identified as tubulin polymerisation inhibitors^19^. Structural modification of the chalcone scaffold of compound **1**, by replacement of the 4′-methoxy-3′-hydoxyphenyl ring by an indole, led to a unique class of indole-substituted chalcone derivatives wherein the aromatic ring system consists of indole and 3′,4′,5′-trimethoxyphenyl moieties. Such compounds have been explored by many research groups who have demonstrated a broad spectrum of promising anticancer activity^20^^,^^21^.

Among the synthesized indole-based chalcone derivatives, Trabbic et al.^22^ reported compound **3**, characterized by the presence of a 5-methoxy-2-methyl indole ring, with moderate antiproliferative activity (IC_50_>10 μM) against the U251 glioblastoma cancer cell line. Kumar et al.^23^ showed that indolyl chalcone with general structure **4** inhibited the growth of the A549, PaCa-2 and PC-3 cancer cell lines at micromolar concentrations.

Among the synthetic antitubulin agents having an indole as their core nucleus, it was observed that the presence of an alkoxycarbonyl function at the 2-position of the indole ring is one of the key building elements of a series of either 2-alkoxycarbonyl-3-[(3′,4′,5′-trimethoxyphenyl)thio]indole or 2-alkoxycarbonyl-3-(3′,4′,5′-trimethoxybenzoyl)indole derivatives with general structure **5** synthesized by De Martino et al. These compounds were characterized by electron-withdrawing (Cl or Br) or electron-donating (OMe) groups at the C-5 position of the indole ring^24–26^. A SAR study revealed that in this series of compounds, based on the 2-alkoxycarbonyl-*N*-1*H*-indole system, derivatives characterized by the presence of a 5-methoxy group on the indole skeleton and by a carbonyl or sulphur linker between the 3-position of the indole nucleus and the 3′,4′,5′-trimethoxyphenyl ring were found to be potent inhibitors of both tubulin polymerisation and MCF-7 human breast carcinoma cell growth^19^.

**Figure 2.**
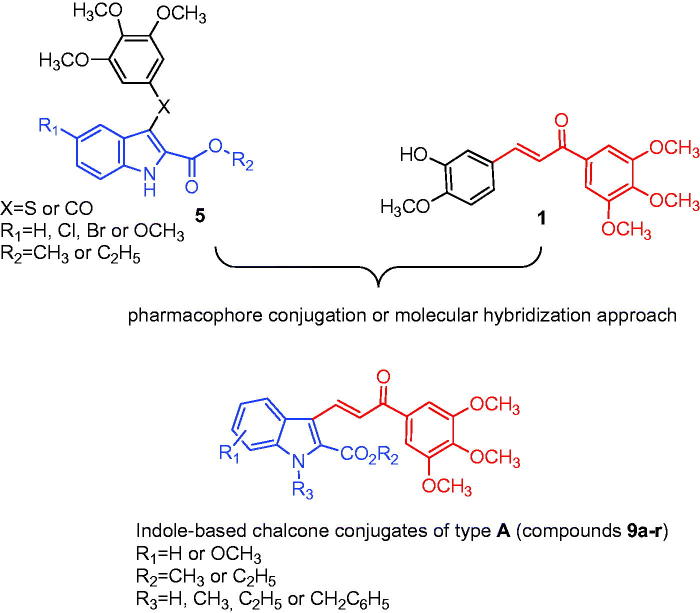
Bioisosteric replacement of the sulphur or carbonyl group (X) of compound **5** with the 2-propen-1-one system (drawn in red) of chalcone **1** furnished a new class of indole-based chalcone conjugates of type A.

Due to the importance of the 1-(3′,4′,5′-trimethoxyphenyl)-2-propen-1-one and the 2-alkoxycarbonyl indole moiety in the antiproliferative and tubulin polymerisation activities of compounds with structures **1** and **5**, respectively, we subsequently combined both features in the chalcone structure by a pharmacophore fusion approach to design a new class of 3-(2′-alkoxycarbonyl substituted indolyl)-1-(3′,4′,5′-trimethoxyphenyl)-prop-2-en-1-one conjugates with general structure of type **A**, corresponding to compounds **9a–r** (Figure 2).

The SAR study on the indole core was investigated by varying (a) the location of the methoxy group (*R*_1_) at the 5- or 6-position; (b) the alkyl group (*R*_2_ = methyl or ethyl) of the electron-withdrawing 2-alkoxycarbonyl group; and (c) the substituent at the *N*-1 position (we selected hydrogen, methyl, ethyl or benzyl to examine the substituent *R*_3_). In this series of indole-based chalcone derivatives, we maintained the 3′,4′,5′-trimethoxyphenyl ring attached to the carbonyl of the 2-propen-1-one system of compound **1**, replacing the second 4′-methoxy-3′-hydoxyphenyl ring with the 2-alkoxycarbonyl indole moiety of derivative **5**, characterized by the presence of various substituents (H, Me, Et or Bn) at the *N* − 1 position as well as a methoxy group at the C-5 or C-6 position of the indole nucleus. Through the synthesis of compounds **9g–j**, **9k–n** and **9o–r**, we investigated whether the introduction of a methyl, ethyl or benzyl moiety, respectively, at the indole nitrogen of derivatives **9a–f** would lead to an increase or loss of activity through a steric effect.

## Materials and methods

### Chemistry-general

^1^H and ^13^ C NMR spectra were recorded on a Bruker AC 200 and Varian 400 Mercury Plus spectrometer, respectively. Chemical shifts (δ) are given in ppm upfield from tetramethylsilane as internal standard, and the spectra were recorded in appropriate deuterated solvents, as indicated. Mass spectra were recorded by an ESI single quadrupole mass spectrometer Waters ZQ 2000 (Waters Instruments, UK) and the values are expressed as [M + 1]^+^. Melting points (mp) were determined on a Buchi–Tottoli apparatus and are uncorrected. All products reported showed ^1^H and ^13^C NMR spectra in agreement with the assigned structures. The purity of tested compounds was determined by combustion elemental analyses conducted by the Microanalytical Laboratory of the Chemistry Department of the University of Ferrara with a Yanagimoto MT-5 CHN recorder elemental analyser. All tested compounds yielded data consistent with a purity of at least 95% as compared with the theoretical values. All reactions were carried out under an inert atmosphere of dry nitrogen. Standard syringe techniques were used for transferring dry solvents. Reaction courses and product mixtures were routinely monitored by TLC on silica gel (pre-coated F_254_ Merck plates), and compounds were visualized with aqueous KMnO_4_. Flash chromatography was performed using 230–400 mesh silica gel and the indicated solvent system. Organic solutions were dried over anhydrous Na_2_SO_4_. ^1^H-NMR and ^13^C-NMR spectra of selected compounds **9a–e**, **9g**, **9l, 9o** and **9q** are included in the Supplementary Material.

### General synthetic procedures

#### General procedure a for the preparation of compounds 6a–f

To a suspension of the appropriate indole-2-carboxylic acid (10 mmol) in methanol or ethanol (30 ml), concentrated sulphuric acid (1 ml) was added and the reaction mixture heated under reflux for 24 h. After the reaction mixture was cooled to room temperature, a 2M Na_2_CO_3_ solution was slowly added (5 ml). The reaction mixture was then concentrated *in vacuo*, and the resulting residue portioned between a saturated NaHCO_3_ solution (10 ml) and EtOAc (40 ml). The organic layer was separated, washed with brine, dried and filtered and the solvent removed under reduced pressure. The crude residue was suspended in a mixture of ethyl ether/light petroleum ether (1:2 v/v, 15 ml), and the suspension was stirred for 10 min. The solid was filtered and used without further purification for the next reaction. For the characterisation of compounds **6a–f** see references^27–31^.

#### General procedure B for the synthesis of compounds 7a–f

DMF (1.62 ml, 21 mmol) was cooled to 0 °C and POCl_3_ (0.55 ml, 6 mmol) was added slowly over 10 min. A solution of the appropriate 2-alkoxycarbonyl-1*H*-indole derivative **6a–f** (5.7 mmol) in DMF (2.0 ml) was added slowly to the cooled solution over a period of 10 min, and the resulting mixture was stirred for 30 min at room temperature and then heated at 60 °C for 4 h until the clear solution became a paste. The reaction mixture was poured into ice-water and basified with 2 M aq Na_2_CO_3_ (3.5 ml) until pH 8 was reached. The precipitate was collected by filtration, washed with cooled water and dried *in vacuo*. Compounds **7a**, **7 b**, **7d** and **7e** are known (see references^29,32,33^).

#### General procedure C for the synthesis of derivatives 8a–l

To a mixture of the appropriate 2-alkoxycarbonyl-3-formyl-1*H*-indole derivative **7a–f** (1 mmol) in *N,N*-dimethylformamide (5 ml) was added sodium hydride (60% in mineral oil, 48 mg, 1.1 mmol, 1.1 equiv.), and the mixture was stirred for 1 h at room temperature. Next, the appropriate alkyl halide (methyl or ethyl iodide) or benzyl bromide (2 mmol, 2 equiv.) was added, and the mixture was stirred at room temperature for 4 h. After this time, saturated aqueous ammonium chloride (5 ml) was added and the organics extracted with dichloromethane (3 × 10 ml). The organic phase was washed with water (10 ml), brine (10 ml), dried and concentrated under reduced pressure. The crude residue was suspended in petroleum ether, the mixture stirred for 15 min and then filtered to afford the corresponding *N*-methyl, *N*-ethyl or *N*-benzyl 2-alkoxycabonyl-3-formyl indole derivatives **8a–d**, **8e–h** and **8i–l**, respectively, which was used for the next reaction without further purification. Compounds **8a**, **8 b** and **8i** are known (see references^32,34,35^).

#### General procedure D for the synthesis of hybrid derivatives 9a–r

In a 25 ml round bottom flask, the appropriate 2-alkoxycarbonyl-3-formylindole **7a–f** or **8a–l** (0.5 mmol), 3,4,5-trimethoxyacetophenone (160 mg, 0.75 mmol, 1.5 equiv.), piperidine (0.080 ml, 64 mg, 0.75 mmol, 1.5 equiv.) and methanol or ethanol as solvent (15 ml) were added, and the reaction mixture was stirred under reflux for 18 h. After this time, the reaction mixture was evaporated to dryness under reduced pressure, the residue suspended in ethyl ether and the mixture stirred for 10 min. The solid was isolated by filtration, rinsed with ethyl ether and dried under vacuum.

*Methyl 3-formyl-1H-indole-2-carboxylate (****7a****).* Following general procedure (B), compound **7a** was isolated as a yellow solid. Yield 78%, mp 199–201 °C. ^1^H-NMR (*d*_6_-DMSO) δ: 3.99 (s, 3H), 7.27 (m, 2H), 7.55 (d, *J* = 7.6 Hz, 1H), 8.23 (d, *J* = 7.6 Hz, 1H), 10.6 (s, 1H), 12.9 (bs, 1H). MS (ESI) *m/z* calculated for C_11_H_9_NO_3_ [M + 1]^+^ = 204.06, found 204.21.

*Ethyl 3-formyl-1H-indole-2-carboxylate (****7b****).* Following general procedure (B), compound **7b** was isolated as a yellow solid. Yield 78%, mp 190–191 °C. ^1^H-NMR (*d*_6_-DMSO) δ: 1.40 (t, *J* = 7.2 Hz, 3H), 4.44 (q, *J* = 7.2 Hz, 2H), 7.29 (m, 2H), 7.56 (d, *J* = 7.4 Hz, 1H), 8.24 (d, *J* = 7.4 Hz, 1H), 10.6 (s, 1H), 12.8 (bs, 1H). MS (ESI) *m/z* calculated for C_12_H_11_NO_3_ [M + 1]^+^ = 218.07, found 218.33.

*Methyl 3-formyl-5-methoxy-1H-indole-2-carboxylate (****7c****).* Following general procedure (B), compound **7c** was isolated as a white solid. Yield 75%, mp 241–243 °C. ^1^H-NMR (*d*_6_-DMSO) δ: 3.78 (s, 3H), 3.95 (s, 3H), 7.02 (dd, *J* = 9.2 and 2.4 Hz, 1H), 7.43 (d, *J* = 9.2 Hz, 1H), 7.67 (d, *J* = 2.4 Hz, 1H), 10.5 (s, 1H), 12.8 (bs, 1H). MS (ESI) *m/z* calculated for C_12_H_11_NO_4_ [M + 1]^+^ = 234.07, found 234.22.

*Ethyl 3-formyl-5-methoxy-1H-indole-2-carboxylate (****7d****).* Following general procedure (B), compound **7d** was isolated as an orange solid. Yield 75%, mp 228–230 °C. ^1^H-NMR (CDCl_3_) δ: 1.47 (t, *J* = 7.2 Hz, 3H), 3.90 (s, 3H), 4.49 (q, *J* = 7.2 Hz, 2H), 7.04 (dd, *J* = 9.0 and 2.4 Hz, 1H), 7.33 (d, *J* = 9.0 Hz, 1H), 7.90 (d, *J* = 2.4 Hz, 1H), 9.43 (bs, 1H), 10.7 (s, 1H). MS (ESI) *m/z* calculated for C_13_H_13_NO_4_ [M + 1]^+^ = 248.08, found 248.36.

*Methyl 3-formyl-6-methoxy-1H-indole-2-carboxylate (****7e****).* Following general procedure (B), compound **7e** was isolated as a brown solid. Yield 63%, mp 148–150 °C. ^1^H-NMR (*d*_6_-CDCl_3_) δ: 3.86 (s, 3H), 3.92 (s, 3H), 6.01 (dd, *J* = 9.6 and 2.4 Hz, 1H), 7.15 (d, *J* = 2.4 Hz, 1H), 7.52 (d, *J* = 9.6 Hz, 1H), 8.72 (bs, 1H), 10.3 (s, 1H). MS (ESI) *m/z* calculated for C_12_H_11_NO_4_ [M + 1]^+^ = 234.07, found 234.33.

*Ethyl 3-formyl-6-methoxy-1H-indole-2-carboxylate (****7f****).* Following general procedure (B), compound **7f** was isolated as a cream-coloured solid. Yield 69%, mp 170–171 °C. ^1^H-NMR (CDCl_3_) δ: 1.39 (t, *J* = 7.0 Hz, 3H), 3.81 (s, 3H), 4.42 (q, *J* = 7.2 Hz, 2H), 6.92 (dd, *J* = 9.2 and 2.4 Hz, 1H), 6.97 (d, *J* = 2.2 Hz, 1H), 8.07 (d, *J* = 9.2 Hz, 1H), 10.6 (s, 1H), 12.6 (bs, 1H). MS (ESI) *m/z* calculated for C_13_H_13_NO_4_ [M + 1]^+^ = 248.08, found 248.40.

*Methyl 3-formyl-1-methyl-1H-indole-2-carboxylate (****8a****).* Following general procedure (C), using iodomethane (284 mg, 0.12 ml) as alkylating agent, compound **8a** was isolated as a brown solid. Yield 91%, mp 142–144 °C. ^1^H-NMR (*d*_6_-DMSO) δ: 4.00 (s, 3H), 4.06 (s, 3H), 7.36 (m, 1H), 7.47 (m, 1H), 7.72 (d, *J* = 8.2 Hz, 1H), 8.28 (d, *J* = 8.0 Hz, 1H), 10.4 (s, 1H). MS (ESI) *m/z* calculated for C_12_H_11_NO_3_ [M + 1]^+^=218.07, found 218.31.

*Ethyl 3-formyl-1-methyl-1H-indole-2-carboxylate (****8b****).* Following general procedure (C), using iodomethane (284 mg, 0.12 ml) as alkylating agent, compound **8b** was isolated as a light brown solid. Yield 88%, mp 113–115 °C. ^1^H-NMR (CDCl_3_) δ: 1.48 (t, *J* = 7.2 Hz, 3H), 4.05 (s, 3H), 4.51 (t, *J* = 7.2 Hz, 2H), 7.36 (m, 2H), 7.44 (m, 1H), 8.50 (d, *J* = 7.8 Hz, 1H), 10.6 (s, 1H). MS (ESI) *m/z* calculated for C_13_H_13_NO_3_ [M + 1]^+^ = 232.09, found 232.25.

*Methyl 3-formyl-5-methoxy-1-methyl-1H-indole-2-carboxylate (****8c****).* Following general procedure (C), using iodomethane (284 mg, 0.12 ml) as alkylating agent, compound **8c** was isolated as a light brown solid. Yield >95%, mp 192–194 °C. ^1^H-NMR (*d*_6_-DMSO) δ: 3.80 (s, 3H), 3.96 (s, 3H), 4.01 (s, 3H), 7.07 (dd, *J* = 9.2 and 2.4 Hz, 1H), 7.64 (d, *J* = 9.2 Hz, 1H), 7.73 (d, *J* = 2.4 Hz, 1H), 10.4 (s, 1H). MS (ESI) *m/z* calculated for C_13_H_13_NO_4_ [M + 1]^+^ = 248.08, found 248.31.

*Methyl 3-formyl-6-methoxy-1-methyl-1H-indole-2-carboxylate (****8d****).* Following general procedure (C), using iodomethane (284 mg, 0.12 ml) as alkylating agent, compound **8d** was isolated as a light brown solid. Yield 88%, mp 101–103 °C. ^1^H-NMR (CDCl_3_) δ: 3.89 (s, 3H), 3.99 (s, 3H), 4.02 (s, 3H), 6.79 (d, *J* = 2.2 Hz, 1H), 6.98 (dd, *J* = 9.0 and 2.2 Hz, 1H), 8.35 (d, *J* = 9.0 Hz, 1H), 10.6 (s, 1H). MS (ESI) *m/z* calculated for C_13_H_13_NO_4_ [M + 1]^+^ = 248.08, found 248.30.

*Methyl 3-formyl-1-ethyl-1H-indole-2-carboxylate (****8e****).* Following general procedure (C), using iodoethane (312 mg, 0.16 ml) as alkylating agent, compound **8e** was isolated as a cream-colored solid. Yield 87%, mp 105–107 °C. ^1^H-NMR (CDCl_3_) δ: 1.48 (t, *J* = 7.0 Hz, 3H), 4.59 (q, *J* = 7.0 Hz, 2H), 4.06 (s, 3H), 7.38 (m, 2H), 7.46 (m, 1H), 8.51 (d, *J* = 7.8 Hz, 1H), 10.6 (s, 1H). MS (ESI) *m/z* calculated for C_13_H_13_NO_3_ [M + 1]^+^ = 232.09, found 232.26.

*Methyl 3-formyl-5-methoxy-1-ethyl-1H-indole-2-carboxylate (****8f****).* Following general procedure (C), using iodoethane (312 mg, 0.16 ml) as alkylating agent, compound **8f** was isolated as a light brown solid. Yield 86%, mp 98–100 °C. ^1^H-NMR (*d*_6_-DMSO) δ: 1.36 (t, *J* = 7.2 Hz, 3H), 3.82 (s, 3H), 3.99 (s, 3H), 4.45 (q, *J* = 7.2 Hz, 2H), 7.07 (dd, *J* = 9.0 and 3.0 Hz, 1H), 7.67 (d, *J* = 9.0 Hz, 1H), 7.76 (d, *J* = 3.0 Hz, 1H), 10.4 (s, 1H). MS (ESI) *m/z* calculated for C_14_H_15_NO_4_ [M + 1]^+^=262.10, found 262.26.

*Ethyl 3-formyl-5-methoxy-1-ethyl-1H-indole-2-carboxylate (****8g****).* Following general procedure (C), using iodoethane (312 mg, 0.16 ml) as alkylating agent, compound **8g** was isolated as a light brown solid. Yield 92%, mp 120–121 °C. ^1^H-NMR (CDCl_3_) δ: 1.43 (m, 6H), 3.90 (s, 3H), 4.52 (m, 4H), 7.02 (d, *J* = 2.4 Hz, 1H), 7.36 (d, *J* = 9.0 Hz, 1H), 7.95 (dd, *J* = 9.0 and 2.4 Hz, 1H), 10.6 (s, 1H). MS (ESI) *m/z* calculated for C_15_H_17_NO_4_ [M + 1]^+^ = 276.12, found 276.45.

*Methyl 3-formyl-6-methoxy-1-ethyl-1H-indole-2-carboxylate (****8h****).* Following general procedure (C), using iodoethane (312 mg, 0.16 ml) as alkylating agent, compound **8h** was isolated as a light brown solid. Yield >95%, mp 83–85 °C. ^1^H-NMR (CDCl_3_) δ: 1.46 (t, *J* = 7.0 Hz, 3H), 3.90 (s, 3H), 4.02 (s, 3H), 4.54 (q, *J* = 7.2 Hz, 2H), 6.80 (d, *J* = 2.2 Hz, 1H), 7.02 (dd, *J* = 8.8 and 2.2 Hz, 1H), 8.36 (d, *J* = 8.8 Hz, 1H), 10.6 (s, 1H). MS (ESI) *m/z* calculated for C_14_H_15_NO_4_ [M + 1]^+^ = 262.10, found 262.41.

*Methyl 3-formyl-1-benzyl-1H-indole-2-carboxylate (****8i****).* Following general procedure (C), using benzyl bromide (342 mg, 0.25 ml) as alkylating agent, compound **8i** was isolated as a white solid. Yield 83%, mp 103–105 °C. ^1^H-NMR (CDCl_3_) δ: 3.98 (s, 3H), 5.83 (s, 2H), 7.04 (m, 2H), 7.29 (m, 1H), 7.42 (m, 5H), 8.54 (dd, *J* = 7.2 and 1.6 Hz, 1H), 10.6 (s, 1H). MS (ESI) *m/z* calculated for C_18_H_15_NO_3_ [M + 1]^+^ = 294.11, found 294.41.

*Ethyl 3-formyl-1-benzyl-1H-indole-2-carboxylate (****8j****).* Following general procedure (C), using benzyl bromide (342 mg, 0.25 ml) as alkylating agent, compound **8j** was isolated as a white solid. Yield 91%, mp 112–114 °C. ^1^H-NMR (CDCl_3_) δ: 1.36 (t, *J* = 7.0 Hz, 3H), 4.38 (t, *J* = 7.0 Hz, 3H), 5.81 (s, 2H), 6.99 (m, 2H), 7.24 (m, 1H), 7.38 (m, 5H), 8.53 (dd, *J* = 7.2 and 1.6 Hz, 1H), 10.7 (s, 1H). MS (ESI) *m/z* calculated for C_19_H_17_NO_3_ [M + 1]^+^ = 308.12, found 308.31.

*Methyl 3-formyl-5-methoxy-1-benzyl-1H-indole-2-carboxylate (****8k****).* Following general procedure (C), using benzyl bromide (348 mg, 0.25 ml) as alkylating agent, compound **8k** was isolated as a brown solid. Yield 86%, mp 120–121 °C. ^1^H-NMR (CDCl_3_) δ: 3.90 (s, 3H), 3.97 (s, 3H), 5.80 (s, 2H), 7.02 (dd, *J* = 9.2 and 2.4 Hz, 1H), 7.26 (m, 6H), 7.98 (d, *J* = 2.4 Hz, 1H), 10.6 (s, 1H). MS (ESI) *m/z* calculated for C_19_H_17_NO_4_ [M + 1]^+^=324.12, found 324.31.

*Methyl 3-formyl-6-methoxy-1-benzyl-1H-indole-2-carboxylate (****8l****).* Following general procedure (C), using benzyl bromide (348 mg, 0.25 ml) as alkylating agent, compound **8l** was isolated as a yellow solid. Yield 73%, mp 86–88 °C. ^1^H-NMR (CDCl_3_) δ: 3.88 (s, 3H), 3.94 (s, 3H), 5.78 (s, 2H), 6.88 (dd, *J* = 9.2 and 2.2 Hz, 1H), 7.14 (d, *J* = 2.2 Hz, 1H), 7.36 (m, 6H), 10.4 (s, 1H). MS (ESI) *m/z* calculated for C_19_H_17_NO_4_ [M + 1]^+^ = 324.12, found 324.29.

*(E)-Methyl 3-(3-oxo-3-(3,4,5-trimethoxyphenyl)prop-1-en-1-yl)-1H-indole-2-carboxylate (****9a****).* Following general procedure (D), using methanol as solvent, compound **9a** was isolated as a yellow solid. Yield 73%, mp 180–183 °C. ^1^H-NMR (*d*_6_-DMSO) δ: 3.75 (s, 3H), 3.89 (s, 6H), 3.94 (s, 3H), 7.28 (t, *J* = 8.0 Hz, 1H), 7.35 (s, 2H), 7.38 (t, *J* = 8.0 Hz, 1H), 7.54 (d, *J* = 8.0 Hz, 1H), 7.78 (d, *J* = 15.6 Hz, 1H), 8.20 (d, *J* = 7.6 Hz, 1H), 8.63 (d, *J* = 15.6 Hz, 1H), 12.6 (bs, 1H). ^13^C-NMR (*d*_6_-DMSO) δ: 52.19, 56.02 (2C), 60.09, 105.87 (2C), 113.22, 116.54, 121.60, 122.19 (2C), 124.77, 125.58, 127.66, 133.38, 136.65, 136.95, 141.49, 152.76 (2C), 161.18, 188.63. MS (ESI) *m/z* calculated for C_22_H_21_NO_6_ [M + 1]^+^ = 396.14, found 396.28. Anal. calcd for C_22_H_21_NO_6_. C, 66.83; H, 5.35; N, 3.54; found: C, 66.68; H, 5.21; N, 3.38.

*(E)-Ethyl 3-(3-oxo-3-(3,4,5-trimethoxyphenyl)prop-1-en-1-yl)-1H-indole-2-carboxylate (****9b****).* Following general procedure (D), using methanol as solvent, compound **9b** was isolated as a yellow solid. Yield 81%, mp 213–214 °C. ^1^H-NMR (*d_6_*-DMSO) δ: 1.30 (t, *J* = 7.2 Hz, 3H), 3.68 (s, 3H), 3.82 (s, 6H), 4.32 (q, *J* = 7.2 Hz, 2H), 7.21 (t, *J* = 8.0 Hz, 1H), 7.28 (s, 2H), 7.32 (t, *J* = 8.0 Hz, 1H), 7.48 (d, *J* = 8.4 Hz, 1H), 7.69 (d, *J* = 16.0 Hz, 1H), 8.14 (d, *J* = 8.0 Hz, 1H), 8.59 (d, *J* = 16.0 Hz, 1H), 12.4 (bs, 1H). ^13^C-NMR (*d_6_*-DMSO) δ: 14.08, 56.01 (2C), 60.08, 61.10, 105.85 (2C), 113.22, 116.38, 121.50, 122.19 (2C), 124.80, 125.54, 127.56, 133.41, 136.63, 137.17, 141.42, 152.76 (2C), 160.80, 188.73. MS (ESI) *m/z* calculated for C_23_H_23_NO_6_ [M + 1]^+^ = 410.15, found 410.29. Anal. calcd for C_23_H_23_NO_6_. C, 67.47; H, 5.66; N, 3.42; found: C, 67.29; H, 5.49; N, 3.28.

*(E)-Methyl 5-methoxy-3-(3-oxo-3-(3,4,5-trimethoxyphenyl)prop-1-en-1-yl)-1H-indole-2-carboxylate (****9c****).* Following general procedure (D), using methanol as solvent, compound **9c** was isolated as a yellow solid. Yield 78%, mp 191–192 °C. ^1^H-NMR (*d_6_*-DMSO) δ: 3.76 (s, 3H), 3.90 (s, 9H), 3.95 (s, 3H), 7.06 (dd, *J* = 8.8 and 2.4 Hz, 1H), 7.35 (s, 2H), 7.38 (d, *J* = 8.8 Hz, 1H), 7.56 (d, *J* = 2.4 Hz, 1H), 7.83 (d, *J* = 16.0 Hz, 1H), 8.67 (d, *J* = 16.0 Hz, 1H), 12.4 (bs, 1H). ^13^C-NMR (*d*_6_-DMSO) δ: 52.13, 54.99, 55.78 (2C), 60.08, 102.33, 105.65 (2C), 114.29, 116.16, 117.11, 120.69, 125.23, 127.65, 131.73, 133.37, 137.03, 141.31, 152.74 (2C), 155.41, 161.11, 188.17. MS (ESI) *m/z* calculated for C_23_H_23_NO_7_ [M + 1]^+^ = 426.15, found 426.22. Anal. calcd for C_23_H_23_NO_7_. C, 64.93; H, 5.45; N, 3.29; found: C, 64.77; H, 5.28; N, 3.17.

*(E)-Ethyl 5-methoxy-3-(3-oxo-3-(3,4,5-trimethoxyphenyl)prop-1-en-1-yl)-1H-indole-2-carboxylate (****9d****).* Following general procedure (C), using ethanol as solvent, compound **9d** was isolated as a yellow solid. Yield 68%, mp 180–182 °C. ^1^H-NMR (*d*_6_-DMSO) δ: 1.37 (t, *J* = 6.8 Hz, 3H), 3.76 (s, 3H), 3.89 (s, 9H), 4.40 (q, *J* = 6.8 Hz, 2H), 7.03 (dd, *J* = 8.8 and 2.4 Hz, 1H), 7.40 (s, 2H), 7.46 (d, *J* = 8.8 Hz, 1H), 7.55 (d, *J* = 2.4 Hz, 1H), 7.81 (d, *J* = 15.6 Hz, 1H), 8.69 (d, *J* = 15.6 Hz, 1H), 12.3 (bs, 1H). ^13^C-NMR (*d*_6_-DMSO) δ: 14.10, 54.97, 55.78 (2C), 60.06, 60.99, 102.33, 105.64 (2C), 114.27, 116.01, 117.03, 120.59, 125.26, 127.96, 131.69, 133.39, 137.22, 141.28, 152.73 (2C), 155.39, 160.72, 188.23. MS (ESI) *m/z* calculated for C_24_H_25_NO_7_ [M + 1]^+^ = 440.16, found 440.27. Anal. calcd for C_24_H_25_NO_7_. C, 65.59; H, 5.73; N, 3.19; found: C, 65.39; H, 5.58; N, 3.04.

*(E)-Methyl 6-methoxy-3-(3-oxo-3-(3,4,5-trimethoxyphenyl)prop-1-en-1-yl)-1H-indole-2-carboxylate (****9e****).* Following general procedure (D), using methanol as solvent, compound **9e** was isolated as a light brown solid. Yield 69%, mp 178–180 °C. ^1^H-NMR (*d*_6_-DMSO) δ: 3.77 (s, 3H), 3.82 (s, 3H), 3.90 (s, 6H), 3.93 (s, 3H), 6.84 (dd, *J* = 9.2 and 2.4 Hz, 1H), 6.97 (d, *J* = 2.4 Hz, 1H), 7.36 (s, 2H), 7.75 (d, *J* = 16.0 Hz, 1H), 8.10 (d, *J* = 9.2 Hz, 1H), 8.62 (d, *J* = 16.0 Hz, 1H), 12.3 (bs, 1H). ^13^C-NMR (*d_6_*-DMSO) δ: 52.04, 55.16, 56.04 (2C), 60.09, 94.59, 105.88 (2C), 113.23, 117.04, 119.02, 121.49, 123.19, 126.64, 133.38, 136.94, 137.97, 141.48, 152.76 (2C), 158.24, 161.11, 188.63. MS (ESI) *m/z* calculated for C_23_H_23_NO_7_ [M + 1]^+^ = 426.15, found 426.27. Anal. calcd for C_23_H_23_NO_7_. C, 64.93; H, 5.45; N, 3.29; found: C, 64.78; H, 5.31; N, 3.11.

*(E)-Ethyl 6-methoxy-3-(3-oxo-3-(3,4,5-trimethoxyphenyl)prop-1-en-1-yl)-1H-indole-2-carboxylate (****9f****).* Following general procedure (D), using ethanol as solvent, compound **9f** was isolated as a light brown solid. Yield 63%, mp 172–174 °C. ^1^H-NMR (*d*_6_-DMSO) δ: 1.37 (t, *J* = 6.8 Hz, 3H), 3.80 (s, 3H), 3.85 (s, 6H), 3.91 (s, 3H), 4.35 (q, *J* = 6.8 Hz, 2H), 6.91 (m, 2H), 7.22 (s, 2H), 7.71 (d, *J* = 16.0 Hz, 1H), 8.09 (d, *J* = 8.4 Hz, 1H), 8.63 (d, *J* = 16.0 Hz, 1H), 12.1 (bs, 1H). ^13^C-NMR (*d*_6_-DMSO) δ: 14.64, 55.68, 56.56 (2C), 60.62, 95.05, 106.40 (2C), 113.70, 115.10, 117.38, 119.58, 121.92, 123.68, 127.51, 132.03, 133.94, 137.55, 138.47, 153.29 (2C), 158.72, 160.64, 188.04. MS (ESI) *m/z* calculated for C_24_H_25_NO_7_ [M + 1]^+^ = 440.16, found 440.40. Anal. calcd for C_24_H_25_NO_7_. C, 65.59; H, 5.73; N, 3.19; found: C, 65.33; H, 5.56; N, 3.01.

*(E)-Methyl 1-methyl-3-(3-oxo-3-(3,4,5-trimethoxyphenyl)prop-1-en-1-yl)-1H-indole-2-carboxylate (****9g****).* Following general procedure (D), using methanol as solvent, compound **9g** was isolated as a yellow solid. Yield 68%, mp 147–149 °C. ^1^H-NMR (*d_6_*-DMSO) δ: 3.75 (s, 3H), 3.88 (s, 6H), 3.95 (s, 3H), 4.00 (s, 3H), 7.33 (m, 3H), 7.46 (t, *J* = 8.4 Hz, 1H), 7.69 (d, *J* = 8.4 Hz, 1H), 7.73 (d, *J* = 16.0 Hz, 1H), 8.21 (d, *J* = 8.4 Hz, 1H), 8.40 (d, *J* = 15.6 Hz, 1H). ^13^C-NMR (*d*_6_-DMSO) δ: 33.02, 52.96, 56.57 (2C), 60.63, 106.41 (2C), 112.16, 117.01, 122.03, 122.69, 123.07, 124.22, 126.15, 130.64, 133.89, 137.58, 139.06, 142.05, 153.30 (2C), 162.01, 189.06. MS (ESI) *m/z* calculated for C_23_H_23_NO_6_ [M + 1]^+^ = 410.15, found 409.85. Anal. calcd for C_23_H_23_NO_6_. C, 67.47; H, 5.66; N, 3.42; found: C, 67.33; H, 5.50; N, 3.28.

*(E)-Ethyl 1-methyl-3-(3-oxo-3-(3,4,5-trimethoxyphenyl)prop-1-en-1-yl)-1H-indole-2-carboxylate (****9h****).* Following general procedure (D), using methanol as solvent, compound **9h** was isolated as a yellow solid. Yield 58%, mp 138–140 °C. ^1^H-NMR (*d*_6_-DMSO) δ: 1.37 (t, *J* = 7.2 Hz, 3H), 3.74 (s, 3H), 3.88 (s, 6H), 4.02 (s, 3H), 4.39 (q, *J* = 7.2 Hz, 2H), 7.34 (m, 3H), 7.44 (t, *J* = 8.0 Hz, 1H), 7.70 (m, 2H), 8.22 (d, *J* = 8.0 Hz, 1H), 8.47 (d, *J* = 16.0 Hz, 1H). ^13^C-NMR (*d*_6_-DMSO) δ: 14.46, 32.94, 56.56 (2C), 60.62, 61.99, 106.40 (2C), 112.15, 116.85, 121.85, 122.73, 123.07, 124.19, 126.11, 130.88, 133.92, 137.81, 139.04, 141.99, 153.30 (2C), 161.52, 189.15. MS (ESI) *m/z* calculated for C_24_H_25_NO_6_ [M]^+^ = 424.17, found 424.33. Anal. calcd for C_24_H_25_NO_6_. C, 68.07; H, 5.95; N, 3.31; found: C, 67.84; H, 5.72; N, 3.20.

*(E)-Methyl 5-methoxy-1-methyl-3-(3-oxo-3-(3,4,5-trimethoxyphenyl)prop-1-en-1-yl)-1H-indole-2-carboxylate (****9i****).* Following general procedure (D), using methanol as solvent, compound **9i** was isolated as a yellow solid. Yield 56%, mp 150–151 °C. ^1^H-NMR (*d*_6_-DMSO) δ: 3.73 (s, 3H), 3.89 (s, 6H), 3.92 (s, 3H), 3.94 (s, 3H), 3.98 (s, 3H), 7.08 (dd, J = 9.2 and 2.0 Hz, 1H), 7.39 (s, 2H), 7.56 (d, *J* = 2.0 Hz, 1H), 7.62 (d, *J* = 9.2 Hz, 1H), 7.77 (d, *J* = 16.0 Hz, 1H), 8.44 (d, *J* = 16.0 Hz, 1H). ^13^C-NMR (*d*_6_-DMSO) δ: 33.22, 52.89, 55.57, 56.32 (2C), 60.60, 102.89, 106.18 (2C), 113.31, 116.52, 117.33, 121.06, 124.54, 130.64, 133.89, 134.26, 137.70, 141.85, 153.26 (2C), 156.18, 161.95, 188.61. MS (ESI) *m/z* calculated for C_24_H_25_NO_7_ [M]^+^ = 440.16, found 440.39. Anal. calcd for C_24_H_25_NO_7_. C, 65.59; H, 5.73; N, 3.19; found: C, 65.38; H, 5.49; N, 3.04.

*(E)-Methyl 6-methoxy-1-methyl-3-(3-oxo-3-(3,4,5-trimethoxyphenyl)prop-1-en-1-yl)-1H-indole-2-carboxylate (****9j****).* Following general procedure (D), using methanol as solvent, compound **9j** was isolated as a light brown solid. Yield 78%, mp 135–136 °C. ^1^H-NMR (*d*_6_-DMSO) δ: 3.75 (s, 3H), 3.86 (s, 6H), 3.88 (s, 3H), 3.92 (s, 3H), 3.97 (s, 3H), 6.93 (dd, *J* = 9.2 and 2.4 Hz, 1H), 7.17 (d, *J* = 2.4 Hz, 1H), 7.33 (s, 2H), 7.68 (d, *J* = 16.0 Hz, 1H), 8.08 (d, *J* = 9.2 Hz, 1H), 8.40 (d, *J* = 16.0 Hz, 1H). ^13^C-NMR (*d*_6_-DMSO) δ: 33.13, 52.73, 55.99, 56.56, 60.63, 94.23, 106.40 (2C), 113.95, 117.71, 118.29, 121.98, 123.62, 129.42, 133.89, 137.20, 137.71, 140.52, 142.02, 153.29 (2C), 159.05, 161.94, 189.05. MS (ESI) *m/z* calculated for C_24_H_25_NO_7_ [M]^+^=440.16, found 440.42. Anal. calcd for C_24_H_25_NO_7_. C, 65.59; H, 5.73; N, 3.19; found: C, 65.41; H, 5.51; N, 3.01.

*(E)-Methyl 1-ethyl-3-(3-oxo-3-(3,4,5-trimethoxyphenyl)prop-1-en-1-yl)-1H-indole-2-carboxylate (****9k****).* Following general procedure (D), using methanol as solvent, compound **9k** was isolated as a yellow solid. Yield 65%, mp 115–117 °C. ^1^H-NMR (*d*_6_-DMSO) δ: 1.32 (t, *J* = 7.6 Hz, 3H), 3.75 (s, 3H), 3.88 (s, 6H), 3.96 (s, 3H), 4.54 (q, *J* = 7.2 Hz, 2H), 7.33 (m, 3H), 7.46 (t, *J* = 8.4 Hz, 1H), 7.72 (d, *J* = 8.4 Hz, 1H), 7.73 (d, *J* = 16.0 Hz, 1H), 8.21 (d, *J* = 8.0 Hz, 1H), 8.38 (d, *J* = 16.0 Hz, 1H). ^13^C-NMR (*d_6_*-DMSO) δ: 15.37, 52.51, 56.03 (2C), 60.09, 105.86 (2C), 111.51, 116.74, 121.76, 122.31, 122.56, 123.91, 125.72, 129.15, 133.35, 137.06, 137.50, 141.49, 152.77 (2C), 161.42, 188.52. MS (ESI) *m/z* calculated for C_24_H_25_NO_6_ [M]^+^ = 424.17, found 424.31. Anal. calcd for C_24_H_25_NO_6_. C, 68.07; H, 5.95; N, 3.31; found: C, 67.88; H, 5.79; N, 3.16.

*(E)-Methyl 1-ethyl-5-methoxy-3-(3-oxo-3-(3,4,5-trimethoxyphenyl)prop-1-en-1-yl)-1H-indole-2-carboxylate (****9l****).* Following general procedure (D), using methanol as solvent, compound **9l** was isolated as a yellow solid. Yield 71%, mp 141–142 °C. ^1^H-NMR (*d*_6_-DMSO) δ: 1.32 (t, *J* = 7.2 Hz, 3H), 3.76 (s, 3H), 3.89 (s, 6H), 3.90 (s, 3H), 3.97 (s, 3H), 4.53 (q, *J* = 7.2 Hz, 2H), 7.10 (dd, *J* = 9.2 and 2.0 Hz, 1H), 7.40 (s, 2H), 7.57 (d, *J* = 2.0 Hz, 1H), 7.66 (d, *J* = 9.2 Hz, 1H), 7.79 (d, *J* = 15.6 Hz, 1H), 8.43 (d, *J* = 15.6 Hz, 1H). ^13^C-NMR (*d*_6_-DMSO) δ: 15.49, 52.41, 55.07, 55.80 (2C), 60.08, 102.53, 105.65 (2C), 112.63, 116.27, 116.89, 117.33, 120.78, 124.37, 128.99, 132.68, 133.34, 137.20, 141.34, 152.73 (2C), 155.67, 161.33, 188.10. MS (ESI) *m/z* calculated for C_25_H_27_NO_7_ [M]^+^ = 454.18, found 454.37. Anal. calcd for C_25_H_27_NO_7_. C, 66.21; H, 6.00; N, 3.09; found: C, 65.98; H, 5.89; N, 2.90.

*(E)-Ethyl 1-ethyl-5-methoxy-3-(3-oxo-3-(3,4,5-trimethoxyphenyl)prop-1-en-1-yl)-1H-indole-2-carboxylate (****9m****).* Following general procedure (D), using ethanol as solvent, compound **9m** was isolated as a yellow solid. Yield 62%, mp 160-161 °C. ^1^H-NMR (*d*_6_-DMSO) δ: 1.31 (t, *J* = 6.8 Hz, 3H), 1.35 (t, *J* = 7.2 Hz, 3H), 3.76 (s, 3H), 3.89 (s, 6H), 3.91 (s, 3H), 4.43 (q, *J* = 7.2 Hz, 2H), 4.53 (q, *J* = 7.2 Hz, 2H), 7.13 (dd, *J* = 8.8 and 2.4 Hz, 1H), 7.40 (s, 2H), 7.58 (d, *J* = 2.4 Hz, 1H), 7.66 (d, *J* = 8.8 Hz, 1H), 7.73 (d, *J* = 16.0 Hz, 1H), 8.50 (d, *J* = 16.0 Hz, 1H). ^13^C-NMR (*d*_6_-DMSO) δ: 13.89. 15.50, 40.21, 55.09, 55.81 (2C), 60.07, 61.42, 102.56, 105.67 (2C), 112.61, 116.16, 116.81, 120.65, 124.35, 129.27, 132.64, 133.36, 137.40, 141.54, 152.74 (2C), 155.66, 160.83, 188.20. MS (ESI) *m/z* calculated for C_26_H_29_NO_7_ [M]^+^ = 468.19, found 468.42. Anal. calcd for C_26_H_29_NO_7_. C, 66.80; H, 6.25; N, 3.00; found: C, 66.62; H, 6.02; N, 2.88.

*(E)-Methyl 1-ethyl-6-methoxy-3-(3-oxo-3-(3,4,5-trimethoxyphenyl)prop-1-en-1-yl)-1H-indole-2-carboxylate (****9n****).* Following general procedure (D), using methanol as solvent, compound **9n** was isolated as a yellow solid. Yield 77%, mp 157–158 °C. ^1^H-NMR (*d*_6_-DMSO) δ: 1.31 (t, *J* = 6.8 Hz, 3H), 3.74 (s, 3H), 3.83 (s, 6H), 3.87 (s, 3H), 3.92 (s, 3H), 4.50 (q, *J* = 6.8 Hz, 2H), 6.93 (dd, J = 8.8 and 2.4 Hz, 1H), 7.17 (d, *J* = 2.0 Hz, 1H), 7.33 (s, 2H), 7.68 (d, *J* = 16.0 Hz, 1H), 8.07 (d, *J* = 9.2 Hz, 1H), 8.38 (d, *J* = 15.6 Hz, 1H). ^13^C-NMR (*d*_6_-DMSO) δ: 15.75, 52.78, 56.04, 56.57 (2C), 60.63, 65.38, 94.07, 106.41 (2C), 113.94, 117.99, 118.52, 122.25, 123.74, 128.40, 133.88, 137.73, 139.48, 142.03, 153.29 (2C), 159.11, 161.86, 189.06. MS (ESI) *m/z* calculated for C_25_H_27_NO_7_ [M]^+^ = 454.18, found 454.46. Anal. calcd for C_25_H_27_NO_7_. C, 66.21; H, 6.00; N, 3.09; found: C, 65.90; H, 5.84; N, 2.93.

*(E)-Methyl 1-benzyl-3-(3-oxo-3-(3,4,5-trimethoxyphenyl)prop-1-en-1-yl)-1H-indole-2-carboxylate (****9o****).* Following general procedure (D), using methanol as solvent, compound **9o** was isolated as a yellow solid. Yield 68%, mp 166–168 °C. ^1^H-NMR (*d*_6_-DMSO) δ: 3.75 (s, 3H), 3.88 (s, 9H), 5.82 (s, 2H), 7.03 (d, *J* = 7.2 Hz, 1H), 7.20 (d, *J* = 7.2 Hz, 1H), 7.25 (t, *J* = 7.2 Hz, 1H), 7.33 (m, 5H), 7.43 (t, *J* = 7.2 Hz, 1H), 7.69 (d, *J* = 8.0 Hz, 1H), 7.80 (d, *J* = 15.6 Hz, 1H), 8.23 (d, *J* = 8.0 Hz, 1H), 8.39 (d, *J* = 15.6 Hz, 1H). ^13^C-NMR (*d*_6_-DMSO) δ: 48.46, 53.01, 56.56 (2C), 60.63, 106.43 (2C), 110.00, 112.54, 118.12, 122.90, 123.27, 124.49, 126.49, 126.71 (2C), 127.76, 129.05 (2C), 129.94, 133.78, 137.33, 138.24, 138.88, 142.09, 153.30 (2C), 161.93, 188.97. MS (ESI) *m/z* calculated for C_29_H_27_NO_6_ [M]^+^ = 486.18, found 486.46. Anal. calcd for C_29_H_27_NO_6_. C, 71.74; H, 5.61; N, 2.88; found: C, 71.59; H, 5.38; N, 2.76.

*(E)-Ethyl 1-benzyl-3-(3-oxo-3-(3,4,5-trimethoxyphenyl)prop-1-en-1-yl)-1H-indole-2-carboxylate (****9p****).* Following general procedure (D), using ethanol as solvent, compound **9p** was isolated as a yellow solid. Yield 73%, mp 128–130 °C. ^1^H-NMR (*d_6_*-DMSO) δ: 1.27 (t, *J* = 7.2 Hz, 3H), 3.77 (s, 3H), 3.90 (s, 6H), 4.35 (q, *J* = 7.2 Hz, 2H), 5.84 (s, 2H), 7.04 (d, *J* = 7.6 Hz, 1H), 7.24 (d, *J* = 7.6 Hz, 1H), 7.29 (t, *J* = 7.6 Hz, 1H), 7.38 (m, 5H), 7.42 (t, *J* = 7.6 Hz, 1H), 7.70 (d, *J* = 8.0 Hz, 1H), 7.81 (d, *J* = 15.6 Hz, 1H), 8.26 (d, *J* = 8.0 Hz, 1H), 8.48 (d, *J* = 15.6 Hz, 1H). ^13^C-NMR (*d*_6_-DMSO) δ: 14.29, 48.38, 56.57 (2C), 60.63, 62.06, 106.44 (2C), 117.96, 118.12, 122.69, 122.89, 123.27, 124.46, 126.44, 126.62 (2C), 127.70, 129.03 (2C), 130.20, 133.81, 137.58, 138.24, 138.84, 142.05, 153.32 (2C), 161.45, 189.05. MS (ESI) *m/z* calculated for C_30_H_29_NO_6_ [M]^+^ = 500.20, found 500.41. Anal. calcd for C_30_H_29_NO_6_. C, 72.13; H, 5.85; N, 2.80; found: C, 71.96; H, 5.67; N, 2.65.

*(E)-Methyl 1-benzyl-5-methoxy-3-(3-oxo-3-(3,4,5-trimethoxyphenyl)prop-1-en-1-yl)-1H-indole-2-carboxylate (****9q****).* Following general procedure (D), using methanol as solvent, compound **9q** was isolated as a yellow solid. Yield 67%, mp 170–171 °C. ^1^H-NMR (*d_6_*-DMSO) δ: 3.74 (s, 3H), 3.87 (s, 6H), 3.88 (s, 3H), 3.89 (s, 3H), 5.79 (s, 2H), 6.99 (d, *J* = 7.2 Hz, 2H), 7.05 (dd, *J* = 9.2 and 2.4 Hz, 1H), 7.19 (m, 3H), 7.40 (s, 2H), 7.57 (d, *J* = 2.4 Hz, 1H), 7.60 (d, *J* = 9.2 Hz, 1H), 7.83 (d, *J* = 15.6 Hz, 1H), 8.42 (d, *J* = 15.6 Hz, 1H). ^13^C-NMR (*d*_6_-DMSO) δ: 48.58, 52.92, 55.62, 56.36 (2C), 60.62, 103.11, 106.25 (2C), 113.67, 117.66, 122.00, 124.97, 126.63 (2C), 127.73, 129.04 (2C), 129.86, 133.77, 134.05, 137.48, 138.32, 141.95, 153.28 (2C), 156.28, 161.87, 188.59. MS (ESI) *m/z* calculated for C_30_H_29_NO_7_ [M]^+^=516.19, found 516.50. Anal. calcd for C_30_H_29_NO_7_. C, 69.89; H, 5.67; N, 2.72; found: C, 69.71; H, 5.33; N, 2.54.

*(E)-Methyl 1-benzyl-6-methoxy-3-(3-oxo-3-(3,4,5-trimethoxyphenyl)prop-1-en-1-yl)-1H-indole-2-carboxylate (****9r****).* Following general procedure (C), using methanol as solvent, compound **9r** was isolated as a yellow solid. Yield 63%, mp 148–150 °C. ^1^H-NMR (*d_6_*-DMSO) δ: 3.77 (s, 3H), 3.81 (s, 6H), 3.86 (s, 3H), 3.90 (s, 3H), 5.82 (s, 2H), 7.02 (dd, *J* = 8.8 and 2.2 Hz, 1H), 7.06 (d, *J* = 6.8 Hz, 1H), 7.24 (m, 3H), 7.29 (m, 2H), 7.37 (s, 2H), 7.77 (d, *J* = 15.6 Hz, 1H), 8.12 (d, *J* = 8.8 Hz, 1H), 8.39 (d, *J* = 15.6 Hz, 1H). ^13^C-NMR (*d_6_*-DMSO) δ: 47.82, 52.21, 55.51, 56.04 (2C), 60.10, 94.24, 105.91 (2C), 113.37, 118.03, 118.33, 122.33, 123.27, 125.80, 126.24 (2C), 127.16, 128.13, 128.50, 133.24, 136.91, 137.85, 139.92, 141.56, 152.77 (2C), 158.72, 161.31, 188.45. MS (ESI) *m/z* calculated for C_30_H_29_NO_7_ [M]^+^ = 516.19, found 516.41. Anal. calcd for C_30_H_29_NO_7_. C, 69.89; H, 5.67; N, 2.72; found: C, 69.69; H, 5.36; N, 2.48.

### Biological assays

#### Cell culture and cytotoxic assays

Human cervix carcinoma (HeLa), human colon carcinoma (HT29), human breast carcinoma (MCF-7) and human lymphoma (HL-60) cells (3–5 × 10^4^ cells) and a serial (five-fold) dilution of the test compounds were added to a 96-well microtiter plate. The cells were incubated for 72–96 h (depending on the tumor cell line) at 37 °C in a humidified 5% CO_2_ atmosphere. At the end of the incubation period, the cells were counted in a Coulter Counter (Coulter Electronics Ltd, Harpenden Herts, UK). The IC_50_ (50% inhibitory concentration) was defined as the concentration of compound that inhibited cell proliferation by 50%. The IC_50_ values represent the average (±standard deviation) of at least two to four independent experiments.

U-937 (human myeloid leukaemia) cells from DSMZ (German Collection of Microorganisms and Cell Cultures, Braunschweig, Germany) and U-937 cells overexpressing human Bcl-2 protein (designated U-937/Bcl-2, donated by Dr Jacqueline Bréard, INSERM U749, Faculté de Pharmacie Paris-Sud., Châtenay-Malabry, France) were cultured in RPMI-1640 medium containing 10% (v/v) heat-inactivated foetal bovine serum, 100 μg/mL streptomycin and 100 U/mL penicillin, incubated at 37 °C in a humidified atmosphere containing 5% CO_2_ as described^36^. The functioning of U-937/Bcl-2 cells was tested using the cytotoxic agent 1-β-d-arabino-furanosylcytosine as previously described^37^. Human peripheral blood mononuclear cells (PBMC) were isolated from heparin-anticoagulated blood of healthy volunteers by centrifugation with Ficoll-Paque Plus (GE Healthcare Bio-Sciences AB, Uppsala, Sweden). PBMC were also stimulated with phytohemagglutinin (2 μg/mL) for 48 h before the experimental treatment. The trypan blue dye exclusion method was used for counting the living/dead cells by a hematocytometer with 95% viability in all experiments. The cytotoxicity of compounds was evaluated by the colorimetric 3-(4,5-dimethyl-2-thiazolyl-)-2,5-diphenyl-2*H*-tetrazolium bromide (MTT) assay as previously described^38^. Briefly, 5 × 10^3^ exponentially growing cells were seeded in 96-well microculture plates with increasing concentrations of compounds for 72 h. After the addition of MTT (0.5 mg/mL), cells were incubated at 37 °C for 4 h. Sodium dodecyl sulphate (10% w/v) in 0.05 M HCl was added to the wells and then incubated at room temperature overnight in the dark. Absorbance was measured at 570 nm. Concentrations inducing 50% inhibition of cell growth (IC_50_) were determined graphically for each experiment by non-linear regression using the curve-fitting routine of the computer software Prism™ 4.0 (GraphPad) and the equation derived by De Lean et al.^39^ Values are mean ± SE from at least three independent experiments, each performed in triplicate. Compounds were dissolved in DMSO and kept in the dark at 25 °C. Before each experiment, the compounds were dissolved in culture media at 37 °C, and the final concentration of DMSO did not exceed 0.3% (v/v).

### Effects on tubulin polymerisation and on colchicine binding to tubulin

To evaluate the effect of the compounds on tubulin assembly *in vitro*^40^, varying concentrations of compounds were preincubated with 10 μM bovine brain tubulin in 0.8M monosodium glutamate (pH adjusted to 6.6 with HCl in a 2.0M stock solution) at 30 °C and then cooled to 0 °C. After addition of 0.4 mM GTP, the mixtures were transferred to 0 °C cuvettes in a recording spectrophotometer and warmed to 30 °C. Tubulin assembly was followed turbidimetrically at 350 nm. The IC_50_ was defined as the compound concentration that inhibited the extent of assembly by 50% after a 20 min incubation. The capacity of the test compounds to inhibit colchicine binding to tubulin was measured as described^41^. The reaction mixtures contained 1 μM tubulin, 5 μM [^3^H]colchicine and 5 μM test compound.

### Fluorescent microscopy analysis and evaluation of apoptosis

Fluorescent microscopy and flow cytometric analysis of propidium iodide-stained cells were performed as described^42^. Briefly, cells were harvested and fixed in 3% paraformaldehyde and incubated at room temperature for 10 min. The fixative was removed, and the cells were washed with PBS, resuspended in 30–50 μL of PBS containing 20 μg/mL bis-benzimide trihydrochloride (Hoechst 33258) and incubated at room temperature for 15 min. Stained nuclei were visualized using a Zeiss fluorescence microscope.

Flow cytometric analysis was carried out using a BD FACSVerse™ cytometer (BD Biosciences, San Jose, CA, USA).

### Statistical analysis

Statistical significance of differences between means of control and treated samples were calculated using Student’s *t*-test. *p* < 0.05 were considered significant.

## Results and discussion

### Synthesis

**Scheme 1.**
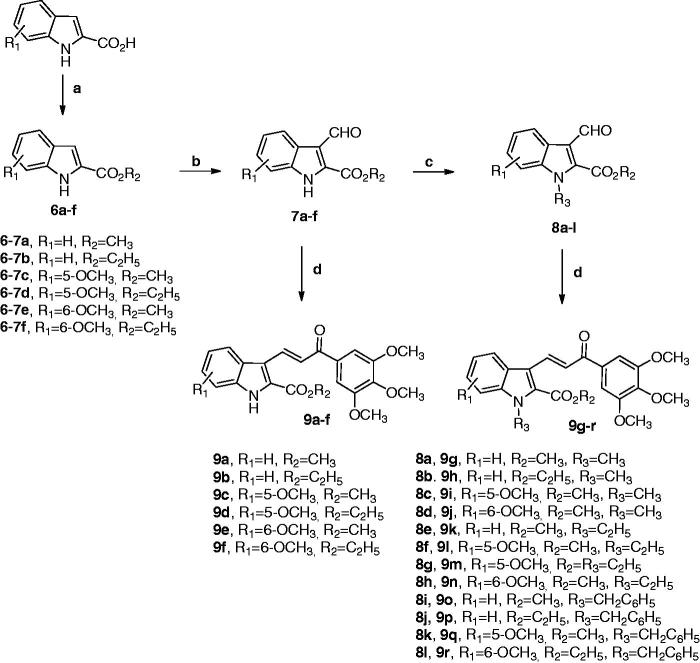
Reagents: (**a)** MeOH or EtOH, conc. H_2_SO_4_, reflux; (**b)** POCl_3_, DMF, 0 °C to rt then Na_2_CO_3_; (**c)** R_3_-halide, NaH, DMF, rt, 2 h; (**d)** 3′,4′,5′-trimethoxyacetophenone, piperidine (cat.), MeOH or EtOH, reflux, 24 h.

Indole-based chalcone derivatives **9a–r** were prepared by the synthetic route outlined in Scheme 1. Indole-2-methyl and ethylcarboxylate **6a–f** were synthesized from the commercially available indole-2-carboxylic acid by esterification using their corresponding alcohol (methanol or ethanol) in the presence of a catalytic amount of H_2_SO_4_. The formylation reaction of 2-alkoxycarbonyl-*N*-1*H*-indole derivatives **6a–f** utilizing Vilsmeier–Haack conditions (POCl_3_ and DMF), followed by workup with a mild base (Na_2_CO_3_), afforded the corresponding 2-alkoxycarbonyl-1*H*-indole-3-carboxaldehydes **7a–f**. *N*-substituted indole aldehydes **8a–l** were synthesized in good yields by the condensation of 2-alkoxycarbonyl-3-formyl-1*H*-indoles **7a–f** with the appropriate alkyl halide (methyl iodide or ethyl iodide) or benzyl bromide using NaH as base in DMF. The subsequent Claisen–Schmidt condensation of *N*-H or *N*-substituted indole aldehydes **7a–f** and **8a–l**, respectively, with 3′,4′,5′-trimethoxyacetophenone in the presence of a catalytic amount of piperidine in refluxing methanol or ethanol as solvent for 24 h, provided the target 2-alkoxycarbonylindole–chalcone derivatives **9a–r**. ^1^H-NMR spectra confirmed the trans (*E*) geometry of the double bond in the enone system of compounds **6a–r**, based on the high values of coupling constants (*J*_TRANS_ = 15–16 Hz) observed between vinylic hydrogens.

### In vitro antiproliferative activities against a panel of four different human cancer cell lines (HeLa, HT29, MCF-7 and HL-60)

With the synthesis of compounds **9a–r**, we focused on the effects on antiproliferative activity obtained by the insertion of the electron-donating methoxy group at the C-5 or C-6 position of the indole ring, combined with the methoxy/ethoxycarbonyl substitution at its C-2 position as well as the presence of a hydrogen, methyl, ethyl or benzyl at the *N* − 1 position of the indole nucleus. We used a panel of four human cancer cell lines, cervix carcinoma (HeLa), colon adenocarcinoma (HT29), breast adenocarcinoma (MCF-7) and promyelocytic leukemia (HL-60) cells. The results are summarized in Table 1, using CA-4 (**2**) and the 2-unsubstituted indolyl chalcone derivative **4a**^23^ as positive controls. All compounds that exhibited an IC_50_ > 20 μM are considered to be inactive for purposes of this discussion. In general, the human promyelocytic leukaemia HL-60 cell line was less sensitive with these compounds as compared with the HeLa, HT29 and MCF-7 cells. In contrast, the HL-60 cells were more sensitive than the other three lines towards the control compound CA-4 (**2**).

**Table 1.** *In vitro* inhibitory effects of compounds **9a–r** and reference compound CA-4 (**2**) and **4a** against the proliferation of human cervix carcinoma (HeLa), human colon adenocarcinoma (HT29), human breast adenocarcinoma (MCF-7) and human promyelocytic leukemia (HL-60) cells.

	IC_50_ (µM)^a^			
Compound	HeLa	HT29	MCF-7	HL-60
**9a**	2.0 ± 1.0	7.3 ± 1.2	1.1 ± 0.4	4.4 ± 0.0
**9b**	2.1 ± 0.8	0.44 ± 0.33	1.8 ± 1.5	26±±13
**9c**	2.4 ± 0.6	1.0 ± 0.7	0.59 ± 0.00	27 ± 1
**9d**	0.33 ± 0.00	0.39 ± 0.05	0.46 ± 0.20	3.4 ± 1.0
**9e**	0.37 ± 0.18	0.16 ± 0.02	0.17 ± 0.03	18 ± 14
**9f**	0.84 ± 0.39	0.13 ± 0.03	0.24 ± 0.14	5.2 ± 2.2
**9g**	69 ± 12	10 ± 4	6.7 ± 3.0	67 ± 46
**9h**	41 ± 11	5.0 ± 1.4	8.3 ± 2.3	58 ± 40
**9i**	25 ± 3	22 ± 1	2.7 ± 0.9	28 ± 3
**9j**	≥ 100	31 ± 22	11 ± 4	14 ± 10
**9k**	9.4 ± 3.1	6.7 ± 1.2	11 ± 10	37 ± 1
**9l**	6.8 ± 2.6	10 ± 6	93 ± 1	26 ± 15
**9m**	24 ± 2	14 ± 6	26 ± 4	19 ± 10
**9n**	24 ± 15	19 ± 9	36 ± 23	47 ± 11
**9o**	8.2 ± 1.5	11 ± 6	71 ± 18	41 ± 25
**9p**	61 ± 41	9.2 ± 4.7	50 ± 4	9.0 ± 5.1
**9q**	15 ± 7	18 ± 10	48 ± 8	39 ± 27
**9r**	26 ± 4	27 ± 2	28 ± 3	32 ± 3
**4a (**nM**)**	81 ± 2	60 ± 3	49 ± 4	23 ± 1
**CA-4 (2,** nM**)**	4 ± 1	3100 ± 200	370 ± 43	1 ± 0.2

^a^IC_50_ compound concentration required to inhibit tumour cell proliferation by 50%. Data are expressed as the mean ± SE from the dose–response curves of at least two to four independent experiments.

The data in Table 1 showed that, among the eighteen synthesized compounds, the most active derivatives were found among the 2-alkoxycarbonyl-*N*-1*H*-indoles **9a–f**, with the C-5 and C-6 methoxy derivatives **9d**, **9e** and **9f** having excellent antiproliferative activity against the HeLa, HT29 and MCF-7 cancer cell lines, with submicromolar IC_50_ values.

Compound **9e**, with the 2-methoxycarbonyl-6-methoxy-*N*-1*H*-indole moiety was the most potent compound of the series, with antiproliferative IC_50_ values of 0.37, 0.16 and 0.17 μM against the HeLa, HT29 and MCF-7 cancer cell lines, respectively. Compounds **9d** and **9f** had nearly equivalent activity, with IC_50_ values lower than 1 μM against these three lines (IC_50_ values of 0.33–0.46 μM and 0.13–0.84 μM, respectively). Furthermore, **9d** and **9f** were significantly more active than **6e** against the HL-60 cells. These data indicate some superiority for the 2-ethoxycarbonyl moiety versus the 2-methoxycarbonyl moiety on the indole, especially because of the generally lower activity of **9c**. In addition, **9d**, **9e** and **9f**, with IC_50_ values in the range 0.13–0.39 μM against the HT29 cell line, were 8–24-fold more potent than CA-4 against this cell line. Compounds **9d**–**f** were similar to CA-4 in their activities against MCF-7 cells (IC_50_s of 0.17–0.46 μM versus 0.37 µM for CA-4). These compounds all contain an indole moiety with no substituent at N-1. The indole chalcone derivative **4a** was three- to five-fold more active than 2-methoxycarbonyl-6-methoxy-*N*-1*H*-indole analogue **9e** against the HeLa, HT29 and MCF-7 cancer cell lines. Moreover, compound **9e** showed substantially reduced activity relative to **4a** against the HL-60 cancer cell line (IC_50_: 18 μM and 23 nM, respectively).

The methoxy substituent on the benzene portion of the indole moiety, as well as the absence or presence of alkyl or benzyl substituents on the indole nitrogen, play an important role in affecting antiproliferative activity. In general, the introduction of an alkyl (methyl or ethyl) or benzyl substituent at the *N*-1 position of the indole ring markedly reduced or eliminated antiproliferative activity, indicating that a steric effect at this position is important for antiproliferative activity, making a hydrogen on the indole nitrogen important for optimal antiproliferative activity.

Further investigation of the SAR showed that antiproliferative activity is also related to the presence of a methoxy group at the C-5 or C-6 position on the benzene portion of the indole ring. For both the 2-methoxycarbonyl and 2-ethoxycarbonyl indole derivatives **9a–f**, compounds with a methoxy group at the C-5 or C-6 position of the indole ring were more potent than the corresponding unsubstituted derivatives **9a** and **9 b**. Moreover, the 6-methoxy analogues **9e** and **9f** were generally more active than the corresponding 5-methoxy counterparts **9c** and **9d**.

For the 5-methoxy-1-*H*-indole analogues **9c** and **9d**, the former methoxycarbonyl derivative **9c** was three- to eight-fold less active than its ethoxycarbonyl counterpart **9d** against HeLa, HT29 and HL-60 tumour cells, but showed similar activity against MCF-7 cells. A contrasting effect was observed comparing the activities of the 6-methoxy-1*H*-indole analogues **9e** and **9f**, with the ethoxycarbonyl derivative **9f** which was 2- and 1.5-fold less active than its methoxycarbonyl indole counterpart **9e** against the HeLa and MCF-7 cancer cell lines, but the two compounds were equipotent against HT-29 tumour cells. Only against the HeLa cancer cell line was **9e** 3.5-fold more active than its ethoxycarbonyl indole homologue **9f**.

For compounds **9c–f**, the position of a methoxy group on the benzene portion of indole moiety was crucial for antiproliferative activity. For the two 2-methoxycarbonyl indole derivatives **9c** and **9e**, a comparison of the substituent effects revealed that the most favourable position for the methoxy substituent was at C-6. The 6-methoxy analogue **9e** was 7-, 6-, 3- and 1.5-fold more potent than its 5-methoxy counterpart **9c** against the HeLa, HT29, MCF-7 and HL-60 cancer cell lines, respectively. We observed a contrasting effect with the 2-ethoxycarbonyl indole analogues **9d** and **9f**, with the 6-methoxy derivative **9f** 2.5- and 1.5-fold less potent than the 5-methoxy isomer **9d** against HeLa and HL-60 cancer cell lines, respectively. Moreover, this latter compound was three- and two-fold less potent than the 6-methoxy isomer **9f** against HT-29 and MCF-7 tumour cells, respectively.

### Inhibition of tubulin polymerisation and colchicine binding

To investigate whether the antiproliferative activities of the C-5 and C-6 methoxy 2-alkoxycarbonyl-*N*-1*H*-indole-based chalcone derivatives **9c–f** derived from an interaction with tubulin, these agents were evaluated for their ability to inhibit tubulin polymerisation and for effects on the binding of [^3^H]colchicine to tubulin (Table 2). For comparison, CA-4 was examined in contemporaneous experiments as a reference compound. The 2-methoxycarbonyl-6-methoxy-*N*-1*H*-indole derivative **9e**, the most potent compound of the series against MCF-7 cells, was found to be also an inhibitor of tubulin polymerisation with an IC_50_ value of 6.2 μM, 10-fold less potent than CA-4 (IC_50_:0.54 μM). The 2-ethoxycarbonylindole homolog **9f**, the most potent derivative of the series against HT-29 cancer cells, weakly inhibited tubulin polymerisation (IC_50_:17 μM) with activity reduced three-fold relative to that of **9e**. Compounds **9c** and **9d** were inactive as inhibitors of tubulin polymerisation, with minimal activity (IC_50_ > 20 μM), although this latter compound demonstrated antiproliferative activity against HeLa cells comparable to that of **9e**. The weak tubulin inhibition suggested the possibility that compounds **9c** and **9d** may possess an additional mechanism of inhibition of HT-29 and MCF-7 cancer cell growth beyond that attributable to the tubulin-based mechanism alone. In particular, when comparing the inhibition of tubulin polymerisation versus the antiproliferative effect, the results demonstrated that the latter activity of compound **9d** against the HeLa, HT29 and MCF-7 cells, with IC_50_ values ranging from 0.33 to 0.46 μM, was in contrast with the weak inhibition of tubulin assembly (IC_50_ > 20 μM) of this derivative. The tumour cell growth assay and the tubulin polymerisation assay differ in a number of important details, such as the tubulin concentration within cells and in the biochemical assembly assay and the presence of different types of microtubule-associated proteins and regulatory proteins expressed in cells but being absent in the biochemical tubulin assay.

**Table 2.** Inhibition of tubulin polymerisation and colchicine binding by compounds CA-4 (**2**) and **9c–f**.

Compound	Tubulin assembly^a^ IC_50_±SD (µM)	Colchicine binding^b^ % ±SD
**9c**	>20	5.3 ± 4
**9d**	>20	9.6 ± 2
**9e**	6.2 ± 0.6	29 ± 5
**9f**	17 ± 2	23 ± 1
CA-4 (**2**)	0.54 ± 0.06	98 ± 0.9

^a^Inhibition of tubulin polymerisation. Tubulin was at 10 µM.

^b^Inhibition of [^3^H]colchicine binding. Tubulin, colchicine and tested compound were at 1, 5 and 5 µM, respectively.

In the colchicine binding studies, compounds **9e** and **9f** at 5 µM displayed 29% and 23%, respectively, inhibition of [^3^H]colchicine binding to tubulin and thus were substantially less potent than CA-4, which in these experiments inhibited colchicine binding by 98% at 5 μM. The data suggested that compounds **9e** and **9f** bind to the colchicine site as their mechanism of inhibition of tubulin polymerisation.

### Compounds 9d, 9e and 9f inhibited proliferation of human leukaemia U-937 and U-937/bcl-2 cells but not normal lymphocytes

**Figure 3.**
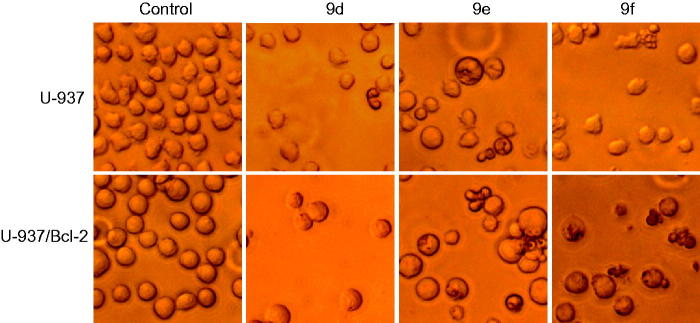
Tumour cells were incubated with vehicle (control), 10 µM (U-937) or 1 μM (U-937/Bcl-2) of the specified compound for 72 h and images were obtained with an inverted phase-contrast microscope.

Compounds **9d**, **9e** and **9f** displayed cytotoxic properties in U-937 cells, as determined by the MTT assay after incubation for 72 h. As with the HL-60 cells, U-937 leukaemia cells were less sensitive relative to the HeLa, HT29 and MCF-7 cells to compounds **9d**, **9e** and **9f**. The IC_50_ values against U-937 cells were 2.9 ± 0.7, 5.7 ± 0.1 and 6.5 ± 1.7 μM for **9d**, **9e** and **9f**, respectively (Table 3). The overexpression of the anti-apoptotic protein Bcl-2 did not confer protection on the cells. Rather, the cell line overexpressing Bcl-2 (U-937/Bcl-2) was more sensitive to the cytotoxic effects of the compounds than the parental cells. The IC_50_ values against U-937/Bcl-2 cells were 0.7 ± 0.2, 1.1 ± 1.0 and 1.0 ± 0.8 μM for **9d**, **9e** and **9f**, respectively. In both cell lines, the three compounds induced a significant reduction in the number of cells, as visualized by phase-contrast microscopy (Figure 3).

**Table 3.** *In vitro* inhibitory effects of compounds **9d, 9e** and **9f** on the proliferation of human leukaemia U-937 and U-937/Bcl-2 cells.

	IC_50_ (μM)^a^	
Compound	U-937	U-937/Bcl-2
**9d**	2.9 ± 0.7	0.7 ± 0.2
**9e**	5.7 ± 0.1	1.1 ± 1.0
**9f**	6.5 ± 1.7	1.0 ± 0.8

^a^IC_50_ compound concentration required to induce 50% inhibition of cell growth. Data are expressed as the mean ± SE from the dose–response curves of at least three independent experiments.

**Figure 4.**
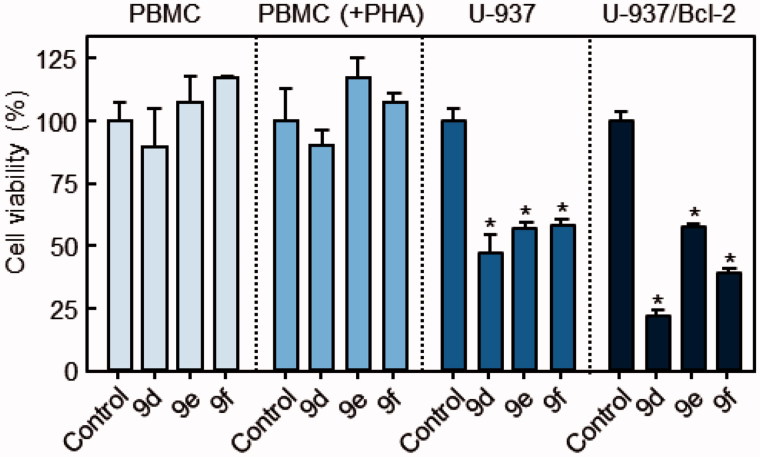
Differential effects of compounds on cell viability of normal PBMCs versus U-937 and U-937/Bcl-2 cells. Human leukaemia, and quiescent and phytohemagglutinin-activated PBMCs [PBMC(+PHA)] from healthy human donors were cultured in the presence of 10 μM of each compound for 24 h. Values represent mean ± SE of three independent experiments each performed in triplicate. *p < 0.05, significantly different from the corresponding control.

By contrast, experiments with normal quiescent or phytohemagglutinine (PHA)-activated healthy human peripheral blood mononuclear cells (PBMC) showed no appreciable toxicity with up to 10 μM **9d**, **9e** or **9f** for 24 h. U-937 and U-937/Bcl-2 cells, which were included in the experiments as positive controls (Figure 4), showed reduced viability in the presence of **9d**, **9e** or **9f**.

### Compounds 9e and 9f induced apoptosis in U-937 and compounds 9d, 9e and 9f induced G_2_-M arrest and apoptosis in U-937/Bcl-2 cells

**Figure 5.**
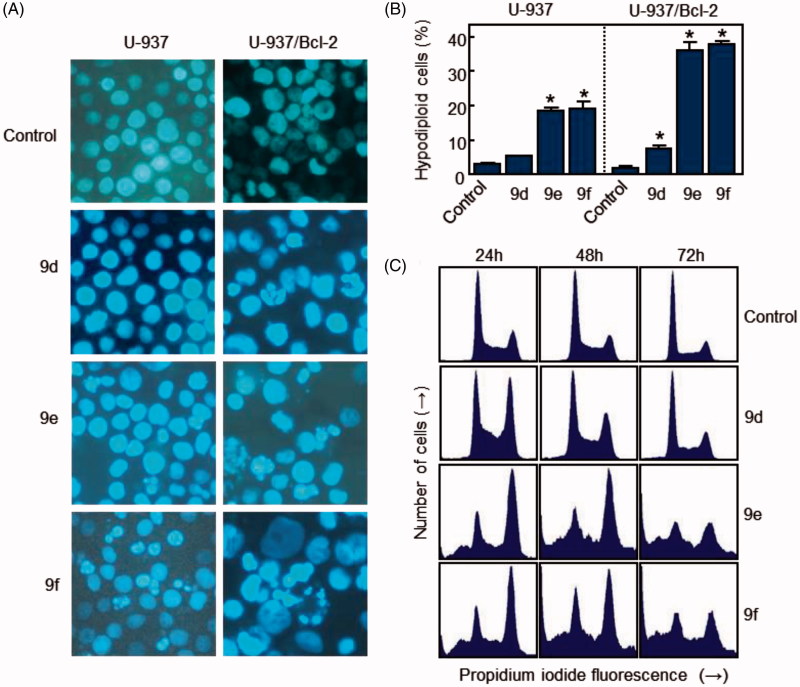
(A) Photomicrographs of representative fields of cells stained with Hoechst 33258 to evaluate nuclear chromatin condensation (i.e. apoptosis) after treatment with 10 μM (U-937) or 1 μM (U-937/Bcl-2) of compounds **9d**, **9e** and **9f** for 72 h. (B) U-937 and U-937/Bcl-2 cells were incubated with 10 or 1 μM, respectively, of the specified compound for 72 h, subjected to flow cytometric analysis using propidium iodide labelling, and the percentage of hypodiploid cells was determined by flow cytometry. Values represent means ± SE from three different experiments performed in triplicate. *p < 0.05, significantly different from control. (C) Representative histograms of flow cytometry after propidium iodide staining of U-937/Bcl-2 cells incubated in the absence or in the presence of 1 μM of the specified compound for 72 h.

To elucidate the molecular mechanism of the cytotoxic effects of **9d**, **9e** and **9f**, we investigated whether these compounds induced the morphological changes characteristic of apoptotic cell death by fluorescence microscopy. Whereas untreated cells exhibited a typically non-adherent, fairly round morphology, cells exposed to **9d**, **9e** and **9f** displayed condensation and fragmentation of chromatin in U-937/Bcl-2 cells. In contrast, compound **9d** did not induce apoptosis in U-937 cells, and it had reduced apoptotic effects in U-937/Bcl-2 cells (Figure 5(A)).

To study effects on the cell cycle, cells were cultured in the presence of the compounds and analysed by flow cytometry. The treatment of U-937/Bcl-2 cells with 1 µM **9e** and **9f** for 24 h caused an arrest of >40% of cells in the G_2_-M phase of the cell cycle, accompanied by a concomitant decrease of cells in the G_1_ and S phases. The percentage of control cells in S phase was ∼27%, which decreased to ∼18% after treatment with compounds **9e** and **9f** for 24 h. The percentage of control cells in G_1_ was ∼43%, which decreased to ∼17% after treatment with compounds **9e** and **9f** for 24 h. The measurement of the number of hypodiploid cells by flow cytometry showed an increase in the percentage of apoptotic cells of 15% (five-fold), 28% (nine-fold) and 36% (∼20-fold) in response to 1 μM **9e** after treatments of 24, 48 and 72 h, respectively (Table 4). Similar increases in the percentage of apoptotic cells were obtained after treatment with 1 μM **9f** for the same incubation times. The fluorescence microscopy and the flow cytometry experiments demonstrated that compound **9d** was not an apoptotic inducer in U-937 cells and only induced low apoptosis in U-937/Bcl-2 cells. Estimates of apoptosis obtained from the number of hypodiploid cells and from Annexin V-FITC staining were similar (data not shown).

**Table 4.** Effect of **9d**, **9e** and **9f** on cell cycle phase distribution of human leukaemia cell cultures.

	µM	% Sub-G_1_	% G_1_	% S	% G_2_-M
U-937					
24 h					
**9d**	0	2.9 ± 0.1	49.0 ± 1.5	23.9 ± 0.8	23.0 ± 0.7
	3	3.5 ± 0.3	52.3 ± 0.2	21.8 ± 0.0	21.2 ± 0.4
	10	5.6 ± 0.5	44.9 ± 0.6	22.9 ± 0.4	24.8 ± 0.3
**9e**	3	6.4 ± 0.4	43.2 ± 0.2	24.4 ± 0.1	24.5 ± 0.1
	10	14.3 ± 1.6*	33.0 ± 2.1*	27.1 ± 0.5	23.2 ± 0.3
**9f**	3	5.9 ± 0.3	42.7 ± 0.5	24.6 ± 0.8	25.2 ± 0.1
	10	11.9 ± 1.6*	36.7 ± 1.8*	25.5 ± 0.5	23.8 ± 0.4
48 h					
**9d**	0	2.9 ± 0.1	54.4 ± 0.4	24.9 ± 0.4	17.2 ± 0.0
	3	2.3 ± 0.1	52.9 ± 0.9	25.8 ± 0.5	18.3 ± 0.3
	10	4.1 ± 0.4	55.3 ± 0.7	23.1 ± 0.1	16.9 ± 0.5
**9e**	3	4.5 ± 0.2	53.6 ± 0.3	24.6 ± 0.3	16.5 ± 0.1
	10	17.9 ± 1.2*	39.7 ± 1.1*	23.3 ± 0.1	17.6 ± 0.0
**9f**	3	5.1 ± 0.1	51.0 ± 0.5	24.0 ± 0.3	19.2 ± 0.5
	10	10.1 ± 0.6*	47.1 ± 1.1*	23.4 ± 0.3	18.2 ± 0.1
72 h					
**9d**	0	7.2 ± 2.0	55.4 ± 2.1	21.6 ± 1.0	14.4 ± 1.5
	3	7.9 ± 0.2	53.6 ± 2.4	21.3 ± 1.0	15.4 ± 1.0
	10	5.4 ± 0.2	55.1 ± 0.0	22.6 ± 0.7	15.5 ± 0.8
**9e**	3	10.8 ± 0.1	52.0 ± 0.9*	21.2 ± 0.7	14.3 ± 0.2
	10	18.4 ± 1.2*	44.5 ± 1.4*	21.4 ± 0.6	14.1 ± 0.4
**9f**	3	6.4 ± 0.6	53.1 ± 2.0	22.3 ± 1.4	16.5 ± 0.6
	10	19.0 ± 3.3*	43.3 ± 1.7	21.2 ± 1.2	15.0 ± 0.6
U-937/Bcl-2					
24 h					
**9d**	0	1.8 ± 0.6	43.2 ± 2.4	27.4 ± 0.2	26.4 ± 1.6
	0.3	2.8 ± 0.2	41.0 ± 1.3	25.1 ± 1.1	29.7 ± 0.2
	1	4.6 ± 0.3	29.3 ± 0.3*	28.7 ± 0.5	35.7 ± 0.2*
**9e**	0.3	9.8 ± 0.5*	24.7 ± 0.5*	23.2 ± 0.2	40.1 ± 1.3*
	1	14.7 ± 0.7*	17.4 ± 0.2*	18.1 ± 1.7*	47.3 ± 1.2*
**9f**	0.3	6.2 ± 0.9	26.0 ± 0.9*	25.2 ± 0.3	41.1 ± 0.4*
	1	20.6 ± 1.6*	17.6 ± 0.6*	18.1 ± 0.1*	41.0 ± 2.3*
48 h					
**9d**	0	3.2 ± 1.1	45.0 ± 3.6	28.6 ± 2.1	22.1 ± 0.2
	0.3	4.4 ± 1.0	49.1 ± 0.4	26.7 ± 2.2	18.9 ± 0.7
	1	6.5 ± 0.5	33.2 ± 2.3*	27.4 ± 0.2	31.0 ± 2.8*
**9e**	0.3	14.6 ± 0.4*	23.0 ± 0.7*	22.4 ± 0.2	34.3 ± 0.5*
	1	28.0 ± 0.7*	13.0 ± 0.9*	15.4 ± 0.1*	33.2 ± 0.9*
**9f**	0.3	11.1 ± 0.1*	27.4 ± 0.8*	26.3 ± 0.4	31.6 ± 0.3*
	1	30.2 ± 3.3*	16.0 ± 0.0*	16.5 ± 1.0*	28.4 ± 1.8*
72 h					
**9d**	0	1.0 ± 0.0	54.3 ± 0.6	21.0 ± 0.3	20.8 ± 0.4
	0.3	3.1 ± 0.9	53.6 ± 1.2	21.8 ± 1.4	20.0 ± 0.8
	1	7.5 ± 1.2	41.6 ± 2.5*	24.0 ± 1.8	24.8 ± 2.6
**9e**	0.3	17.6 ± 0.5*	22.4 ± 0.4*	21.8 ± 0.8	31.3 ± 1.3*
	1	36.1 ± 3.3*	12.8 ± 0.5*	16.0 ± 0.7	23.1 ± 2.2
**9f**	0.3	11.8 ± 0.5*	30.3 ± 2.9*	23.8 ± 0.7	29.4 ± 2.0*
	1	37.7 ± 1.5*	14.0 ± 0.6*	17.1 ± 1.4	22.7 ± 0.2

Cells were cultured with the specified concentrations of **9d**, **9e** and **9f** for the indicated periods of time, and the cell cycle phase distribution was determined by flow cytometry. The values are mean ± SE of two independent experiments with three determinations in each. Asterisks indicate a significant difference (*p* < 0.05) compared to the corresponding controls.

Our data clearly demonstrate that these compounds induce cell death in U-937 as well as in U-937/Bcl-2 cells and, surprisingly, these cells were more sensitive than the parental U-937 cell line. This finding is relevant and of great therapeutic importance because increased expression of Bcl-2 has been associated with chemoresistance, especially in the case of hematologic malignancies^43^. Bcl-2 protein is known to inhibit apoptosis by regulating mitochondrial membrane potential and cytochrome *c* release needed for the activation of caspase-9^44^. We have performed additional experiments with U-937 and U-937/Bcl-2 cells to determine whether the broad spectrum caspase inhibitor Z-VAD-FMK (*N*-benzyloxycarbonyl-Val-Ala-Asp(O-Me) fluoromethyl ketone) was able to block the cell death triggered by compounds **9e** and **9f** (results not shown). Apoptosis was not prevented by Z-VAD-FMK, indicating that these compounds induce cell death by a caspase-independent mechanism.

The fact that Bcl-2 is unable to prevent cytotoxicity suggests that these compounds trigger an alternative pathway that bypasses the mitochondria or that they are able to inactivate the protection conferred by this anti-apoptotic protein. Our results suggest that mitochondria do not play an important role in the induction of cell death. One possibility is that the activation of the extrinsic apoptotic pathway could be responsible for cell death triggered by these compounds in Bcl-2 over-expressing cells, but this is ruled out due to the lack of effect of the broad spectrum caspase inhibitor. Another possibility could be inactivation of Bcl-2 by hyperphosphorylation. In this regard, previous studies have shown that Bcl-2 phosphorylation universally occurs at the G_2_-M phase of the cell cycle^45–47^, and, in addition, this post-translational modification inhibits its prosurvival function^48^^,^^49^. Further studies are needed to determine the effect of these compounds on G_2_-M cell cycle regulators such as B-type cyclin isoforms, cyclin dependent kinase 1, cdc25C and p21^WAF/Cip1^, as well as the potential phosphorylation and inactivation of Bcl-2.

**Figure 6.**
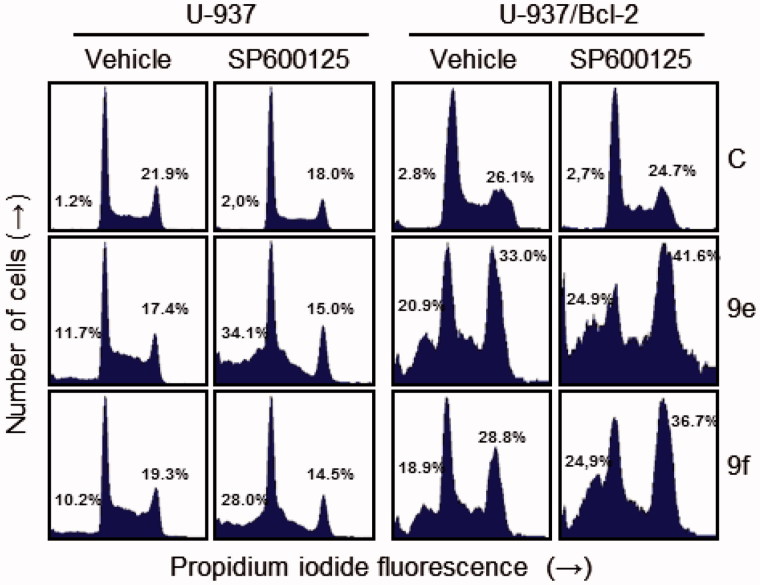
Effects of JNK/SAPK inhibitor on cell cycle distribution in U-937 and U-937/Bcl-2. Cells were pre-treated with SP600125 (10 μM) for 1 h and then treated with the indicated compounds (10 μM in U-937 or 1 μM in U-937/Bcl-2) for 24 h and subjected to flow cytometry using propidium iodide staining. A representative histogram is shown. Values indicate the percentage of cells in sub-G_1_ (left) and G_2_-M phase (right) of the cell cycle.

Additional experiments were performed to determine the involvement of c-Jun-N-terminal kinases/stress-activated protein kinases (JNK/SAPK) in the mechanism of cytotoxicity of these compounds. JNK/SAPK have been implicated in stress-induced apoptosis^50^, but may also be a prosurvival signal. To this end, cells were treated with the JNK/SAPK inhibitor SP600125 (1,9-pyrazoloanthrone) alone or in combination with compounds **9e** and **9f** (Figure 6). We found that SP600125 treatment alone did not affect the percentage of hypodiploid cells, whereas pre-treatment of U-937 cells with the JNK/SAPK inhibitor SP600125 (10 μM) enhanced cell death induced by **9e** and **9f**. Interestingly, inhibition of JNK/SAPK was found to increase G_2_-M cell cycle arrest induced by both compounds in the combination group (**9e**+SP600125 or **9f**+SP600125) in U-937/Bcl-2 cells. These data show that inhibition of JNK/SAPK sensitizes U-937 cells to apoptosis and enhances G_2_-M arrest in U-937/Bcl-2 cells, and these protein kinases might be at least one of the potential targets in the mechanism of signal transduction pathways triggered by these compounds.

## Conclusions

The biological importance of microtubules makes them an interesting target for the development of novel anticancer drugs. Several small synthetic molecules based on the chemical structure of the indole molecular skeleton were found to be effective as inhibitors of tubulin polymerisation. Indole-based chalcone derivatives bearing the trimethoxyphenyl moiety and the indole scaffold were previously reported by several research groups. As a part of our research for novel antitubulin polymerisation agents, we designed and synthesized a novel series of 2-alkoxycarbonyl indole derivatives based on the chalcone scaffold. The most active of these compounds had antiproliferative activities with submicromolar IC_50_s. For our synthetic studies, we choose the 1-(3′,4′,5′-trimethoxyphenyl)-2-propen-1-one moiety of the chalcone system because it was an important pharmacophore of synthetic chalcone derivative **1** previously found to possess antimitotic activity as a colchicine site microtubule destabilizing agent. In our new compounds, we replaced the 3′-hydroxy-4′-methoxyphenyl ring with a 2-alkoxycarbonyl indole. These new indole-inspired chalcone analogues possessed a 2-propen-1-one linker attached to *N*-H, *N*-alkyl or *N*-benzyl-2-alkoxycarbonyl indole ring and a 3′,4′,5′-trimethoxyphenyl ring to produce a series of 1-(3′,4′,5′-trimethoxyphenyl)-3-indolyl-2-propen-1-ones with general structure **A**. The results presented in Table 1 indicate that inhibition of cell growth was highly dependent upon the presence and position of the methoxy substitution on the benzene portion of the indole nucleus as well as the absence of alkylation or benzylation of the *N*-1 position of the indole skeleton. In general, the antiproliferative activities of the compounds was lower against HL-60 cells as compared with the HeLa, HT29 and MCF-7 cancer cell lines.

The 2-methoxycarbonyl and 2-ethoxycarbonyl-6-methoxy-1*H*-indole derivatives **9e** and **9f**, respectively, along with the 2-ethoxycarbonyl-5-methoxy-1*H*-indole analogue **9d**, were the most potent compounds identified in this study, showing potent growth inhibitory activities on HeLa, HT29 and MCF-7 cells, with IC_50_ values in the range 0.13–0.84 μM.

The same derivatives were 8–24-fold more potent than CA-4 against HT29 cancer cell proliferation. Compounds **9d**, **9e** and **9f** were equipotent (**9d**) or 1.5–2-fold more potent (**9e** and **9f**) than CA-4 against MCF-7 cells, but they were much less active than CA-4 against HeLa and HL-60 cells. Our data demonstrated that the antiproliferative activity of compound **9e** was related to inhibition of tubulin polymerisation through an interaction at the colchicine site, although compound **9e** was 10-fold less potent than CA-4 as an inhibitor of tubulin assembly.

In the series of *N*-1H indole derivatives, for the 2-methoxycarbonyl and 2-ethoxycarbonyl indole analogues **9a** and **9 b**, respectively, the introduction of a methoxy group either at the C-5 or C-6 position of the indole nucleus, to afford derivatives **9c-f**, resulted in an increase in antiproliferative activity against the HeLa, HT29 and MCF-7 cancer cells. As an example, among the 2-methoxycarbonyl-*N*-unsubstituted indole analogues **9a**, **9c** and **9e**, the 6-methoxy derivative **9e** was the most active compound against the HeLa, HT-29 and MCF-7 cell lines.

For the 2-methoxycarbonyl-*N*-1*H*-indole derivative **9c**, shifting the methoxy group from the C-5 to the C-6 position, corresponding to compound **9e**, caused 3–6-fold increased activity against HeLa, HT29 and MCF-7 cells, with antiproliferative activities at submicromolar IC_50_s, while the two compounds showed modest activity against HL-60 cells (IC_50_: 27 and 18 μM, respectively).

Comparing the 5-methoxy-1*H*-indole derivatives **9c** and **9d**, the ethoxycarbonyl was superior to the methoxycarbonyl substituent in three of the four cancer cell lines, except the MCF-7 cells, which were equally sensitive to both compounds. A contrasting effect was observed for the 6-methoxy-1*H*-indole derivatives **9e** and **9f**, with the methoxycarbonyl derivative **9e** being 2- and 1.5-fold more potent than its ethoxycarbonyl counterpart **9f** against HeLa and MCF-7 cells, respectively, while this latter compound was 3.5-fold more active than the former against HL-60 cells. These two derivatives were equipotent against the HT-29 cell line.

The SAR studies on these analogues can be summarized by stating that the introduction of a substituent (methyl, ethyl or benzyl) at the *N*-1 position of the indole ring had a negative impact on antiproliferative activity, with the *N*-unsubstituted indole analogs **9a–f** more active than their *N*-substituted counterparts **9g–r** against the HeLa, HT29 and MCF-7 cancer cell lines.

We also demonstrated that the synthetic compounds **9d**, **9e** and **9f** were devoid of significant cytotoxic activity in quiescent or proliferating human PBMCs. These compounds also displayed cytotoxic properties (IC_50_ values ∼1 μM) against the U-937 cell line overexpressing human Bcl-2 (U-937/Bcl-2) via cell cycle progression arrest at the G_2_-M phase and induction of apoptosis. The order of potency of apoptosis induction in U-937/Bcl-2 cells was **9e**–**9f** > **9d**.

## Supplementary Material

Supplemental Material
