# Supplementary material for: Design, synthesis, in vitro antiproliferative activity and apoptosis-inducing studies of 1-(3′,4′,5′-trimethoxyphenyl)-3-(2′-alkoxycarbonylindolyl)-2-propen-1-one derivatives obtained by a molecular hybridisation approach
Source: J Enzyme Inhib Med Chem. 2018 Aug 24;33(1):1225–38. doi: 10.1080/14756366.2018.1493473 (PMC6116705; doi:10.1080/14756366.2018.1493473)
Supplement: Supplemental Material [file IENZ_A_1493473_SM0492.pdf]

Design, synthesis, *in vitro* antiproliferative activity and apoptosis-inducing studies of 1-(3',4',5'-trimetoxyphenyl)-3-(2'-alkoxycarbonylindolyl)-2-propen-1-one derivatives obtained by a molecular hybridization approach

Romeo Romagnoli[a], Delia Preti*[a], Salvatore Pacifico[a], Riccardo Rondanin[a], Barbara Cacciari[a], Ernest Hamel[b], Jan Balzarini[c], Sandra Liekens[c], Dominique Schools[c], Francisco Estévez-Sarmiento[d], José Quintana[d], Francisco Estévez[d]

[a]Dipartimento di Scienze Chimiche e Farmaceutiche, Università di Ferrara, 44121 Ferrara, Italy; [b]Screening Technologies Branch, Developmental Therapeutics Program, Division of Cancer Treatment and Diagnosis, Frederick National Laboratory for Cancer Research, National Cancer Institute, National Institutes of Health, Frederick, Maryland, USA; [c]Rega Institute for Medical Research, KU Leuven, Laboratory of Virology and Chemotherapy, Leuven, Belgium;[d]Departamento de Bioquímica y Biología Molecular, Instituto Universitario de Investigaciones Biomédicas y Sanitarias, Universidad de las Palmas de Gran Canaria, Spain

Supplementary data

^{1}H-NMR and ^{13}C-NMR spectra of compound **9a** 2

^{1}H-NMR and ^{13}C-NMR spectra of compound **9b** 3

^{1}H-NMR and ^{13}C-NMR spectra of compound **9c** 4

^{1}H-NMR and ^{13}C-NMR spectra of compound **9d** 5

^{1}H-NMR and ^{13}C-NMR spectra of compound **9e** 6

^{1}H-NMR and ^{13}C-NMR spectra of compound **9g** 7

^{1}H-NMR and ^{13}C-NMR spectra of compound **9l** 8

^{1}H-NMR and ^{13}C-NMR spectra of compound **9o** 9

^{1}H-NMR and ^{13}C-NMR spectra of compound **9q** 10

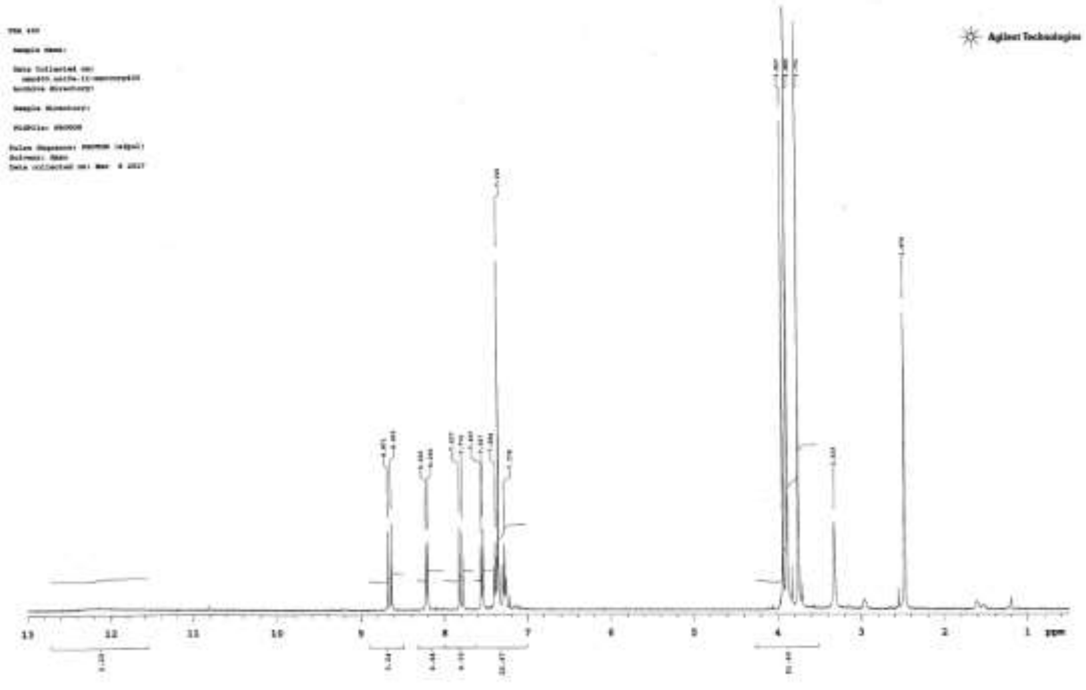

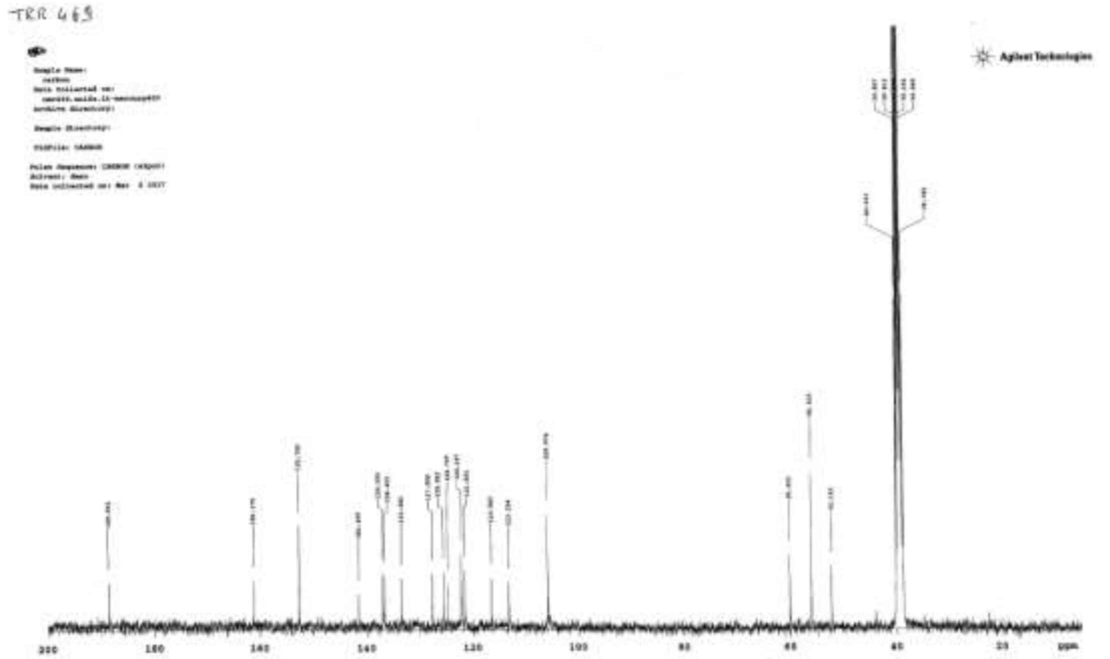

^{1}H-NMR (400 MHz, $CDCl_3$) and ^{13}C-NMR (100 MHz, $CDCl_3$) spectra of compound **9a**

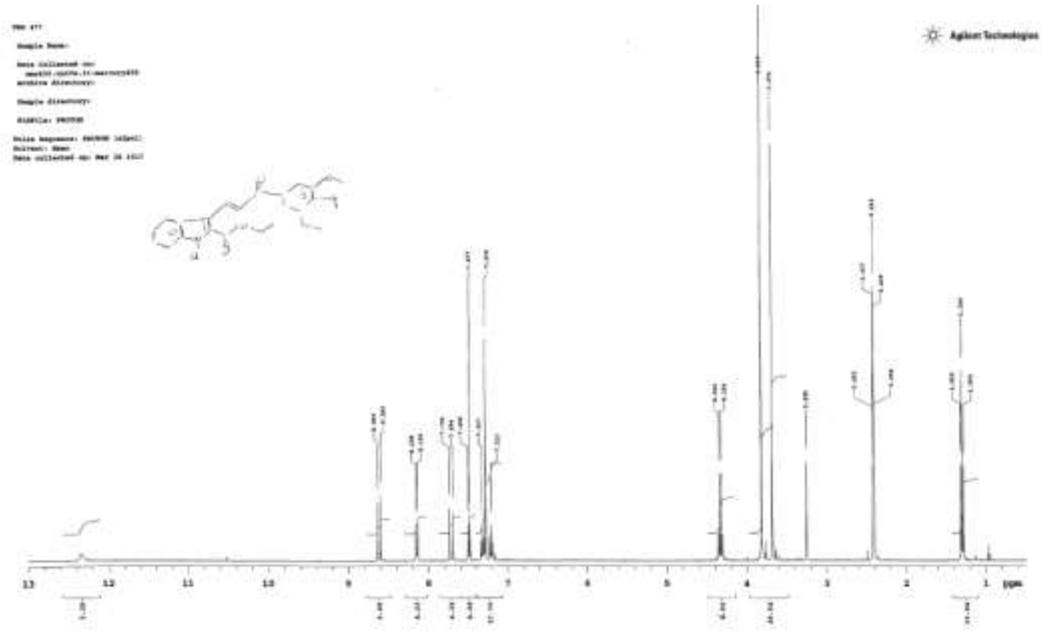

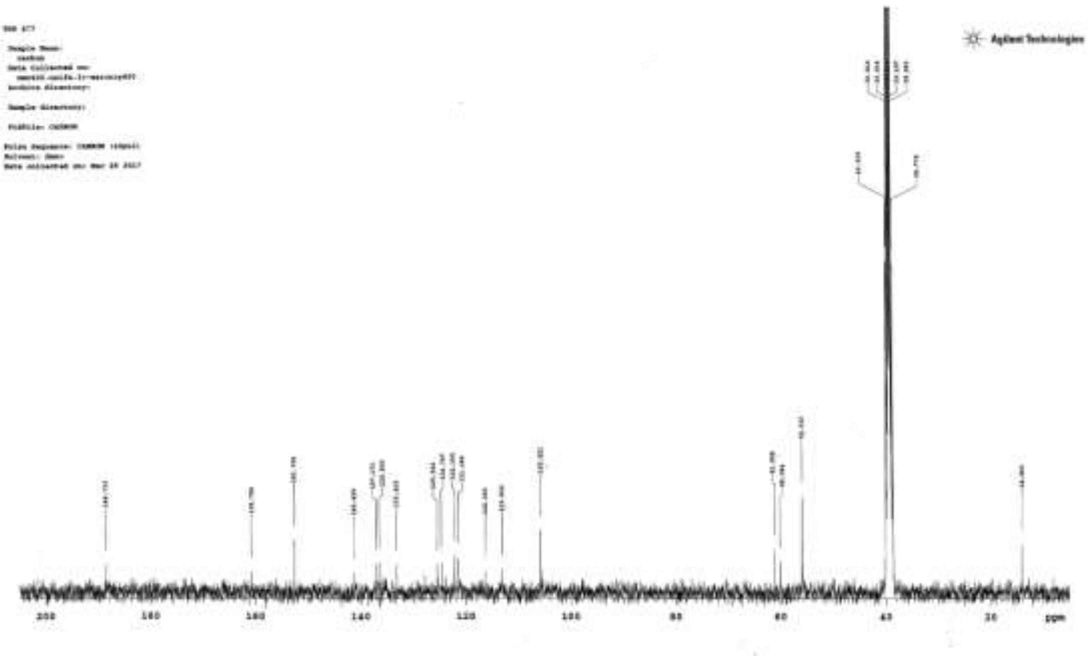

^{1}H-NMR (400 MHz, $CDCl_3$) and ^{13}C-NMR (100 MHz, $CDCl_3$) spectra of compound **9b**

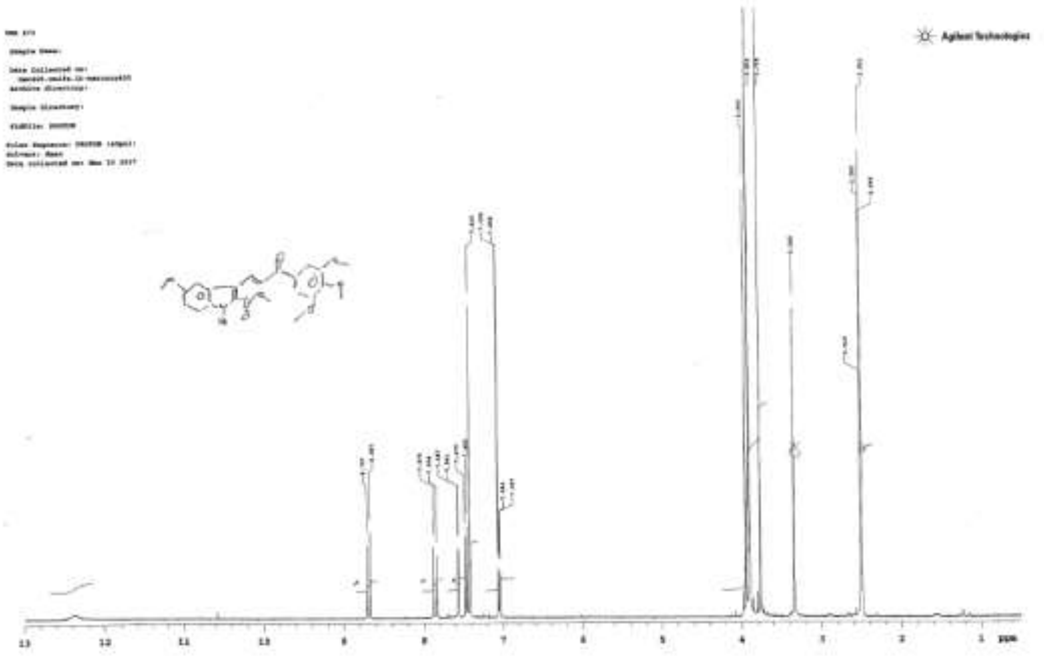

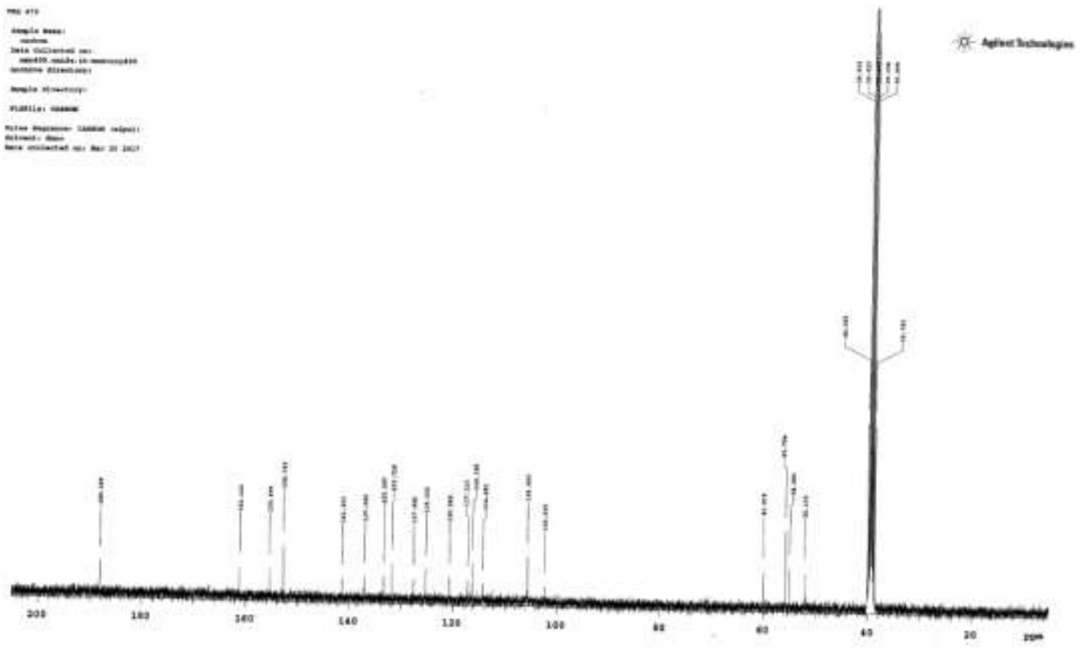

^{1}H-NMR (400 MHz, $CDCl_3$) and ^{13}C-NMR (100 MHz, $CDCl_3$) spectra of compound **9c**

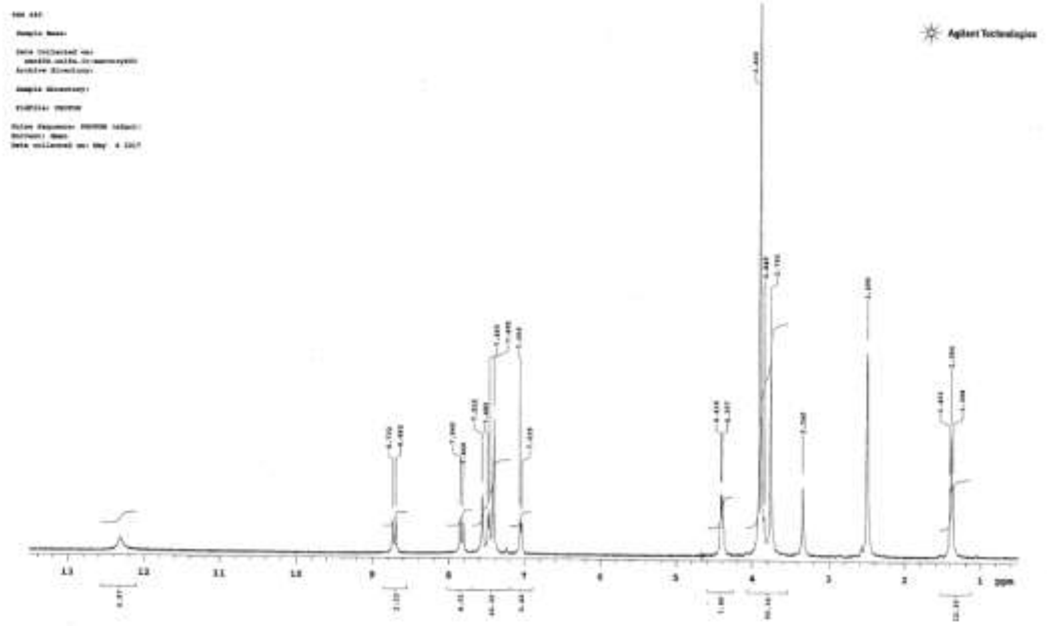

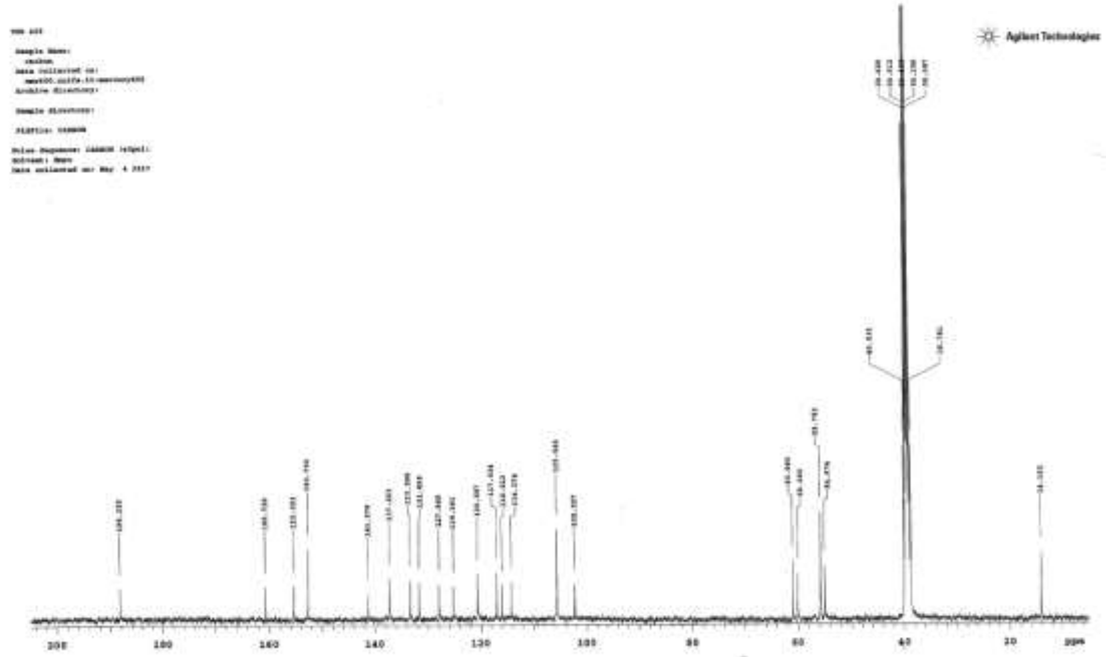

^{1}H-NMR (400 MHz, $CDCl_3$) and ^{13}C-NMR (100 MHz, $CDCl_3$) spectra of compound **9d**

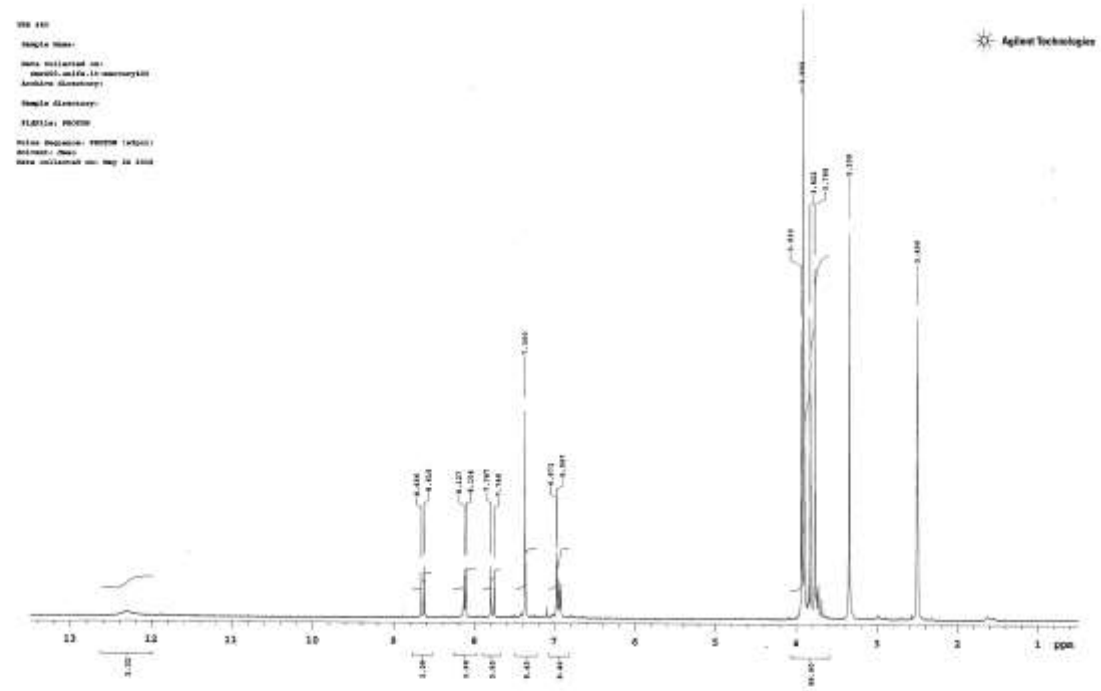

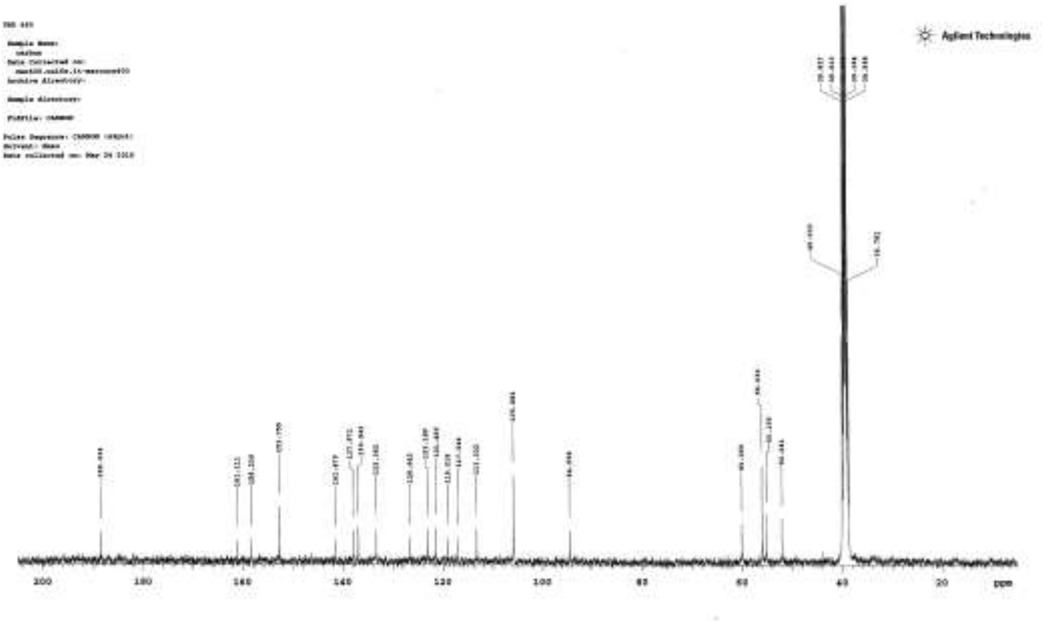

^{1}H-NMR (400 MHz, $CDCl_3$) and ^{13}C-NMR (100 MHz, $CDCl_3$) spectra of compound **9e**

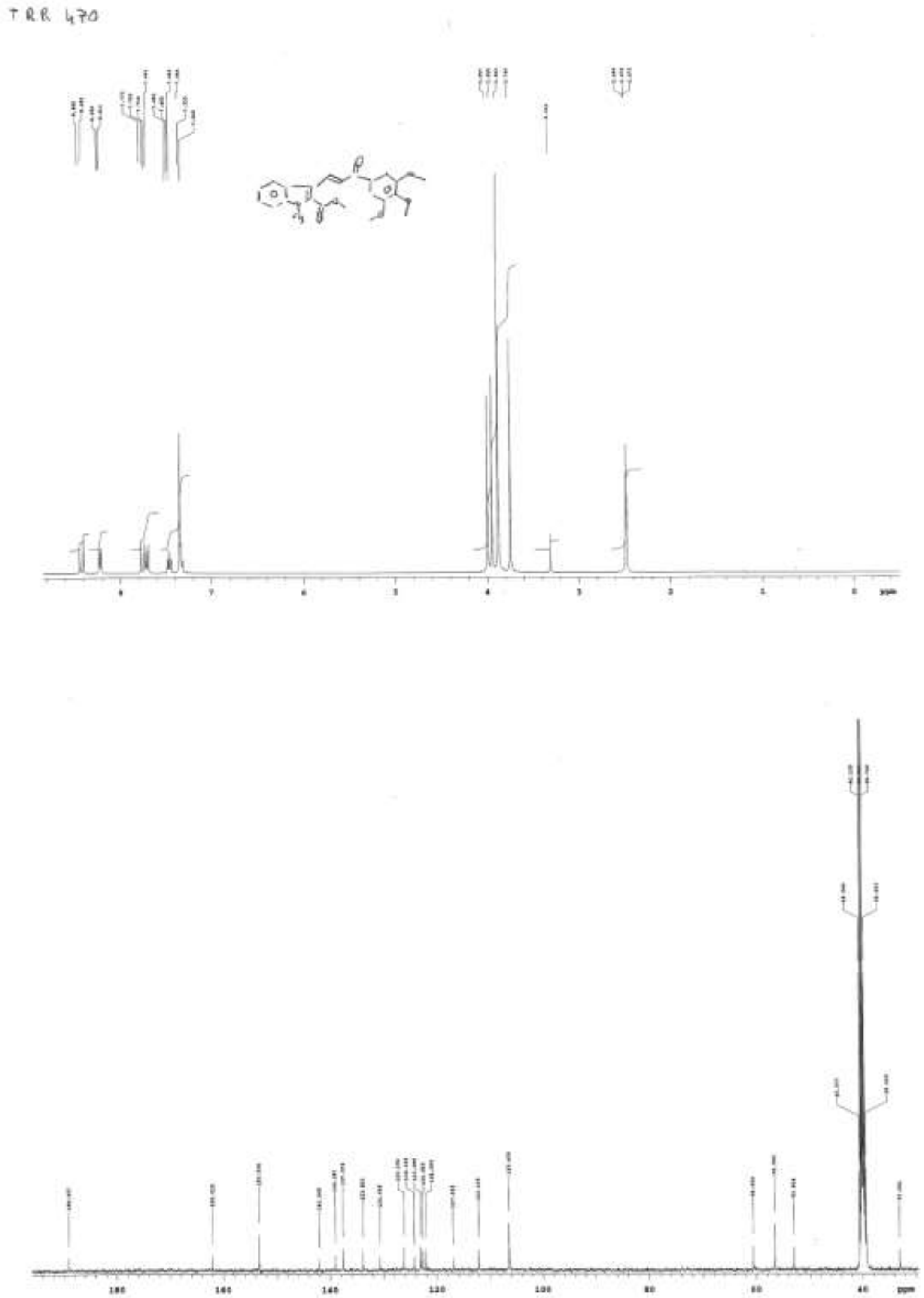

^{1}H-NMR (400 MHz, $CDCl_3$) and ^{13}C-NMR (100 MHz, $CDCl_3$) spectra of compound **9g**

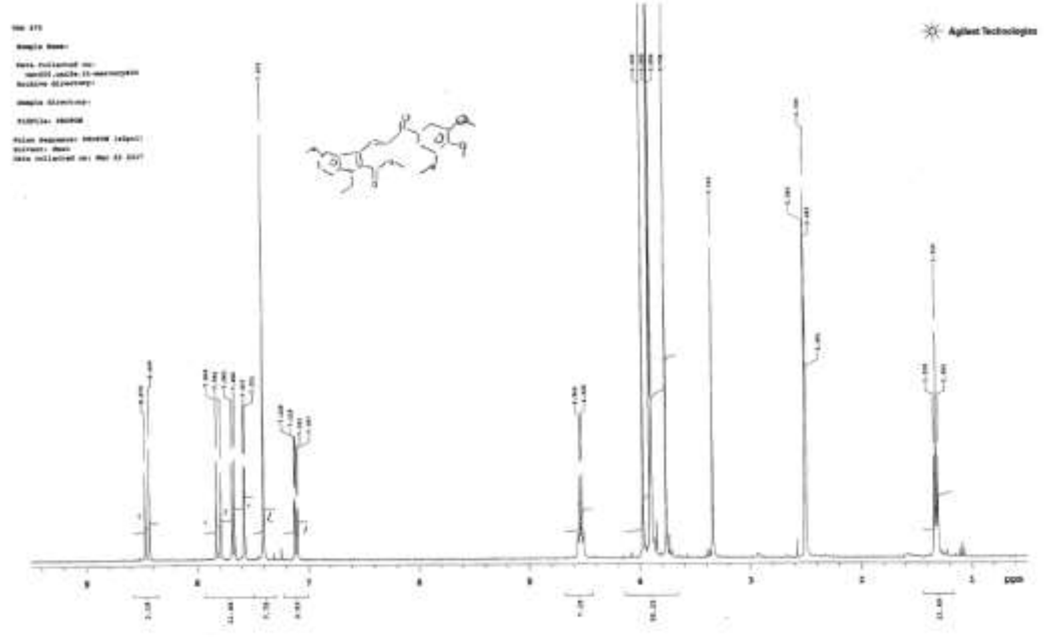

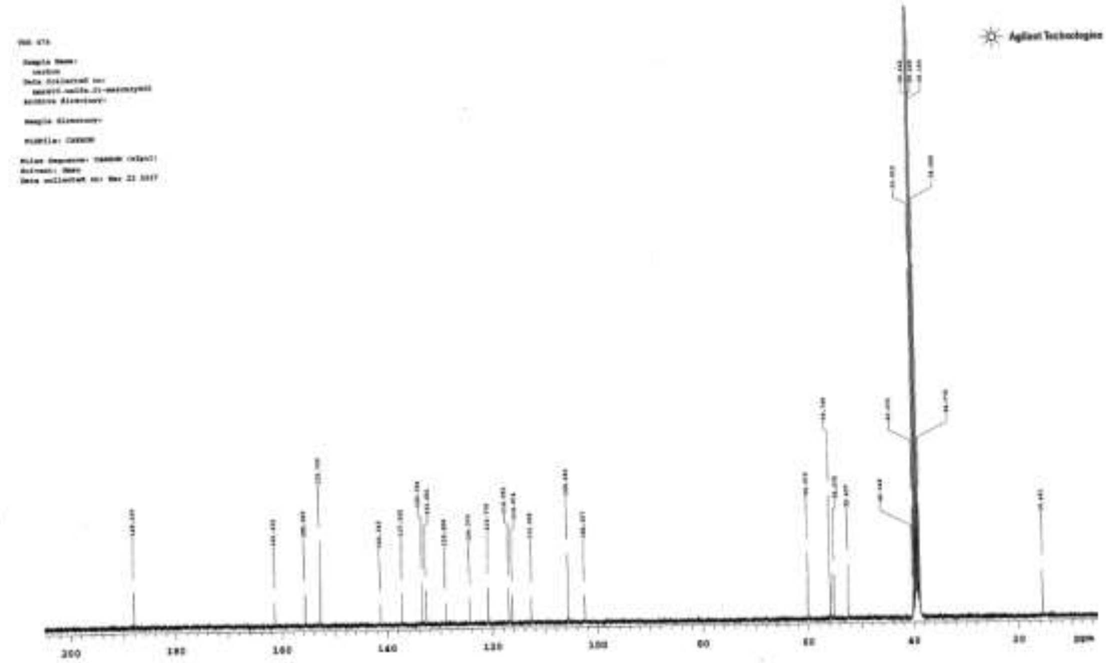

^{1}H-NMR (400 MHz, $CDCl_3$) and ^{13}C-NMR (100 MHz, $CDCl_3$) spectra of compound **9l**

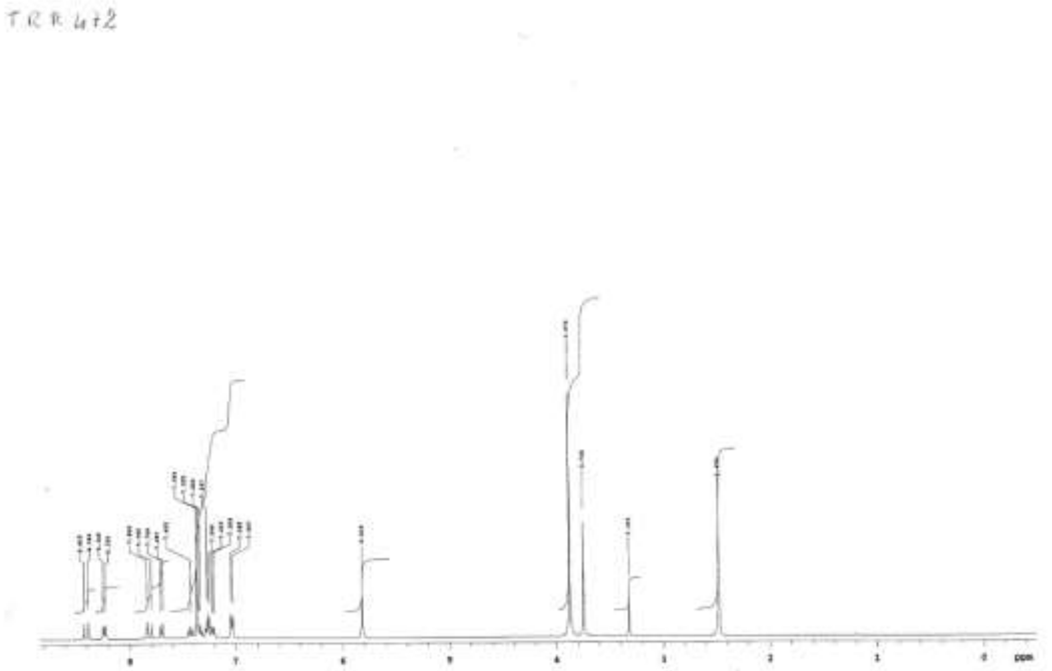

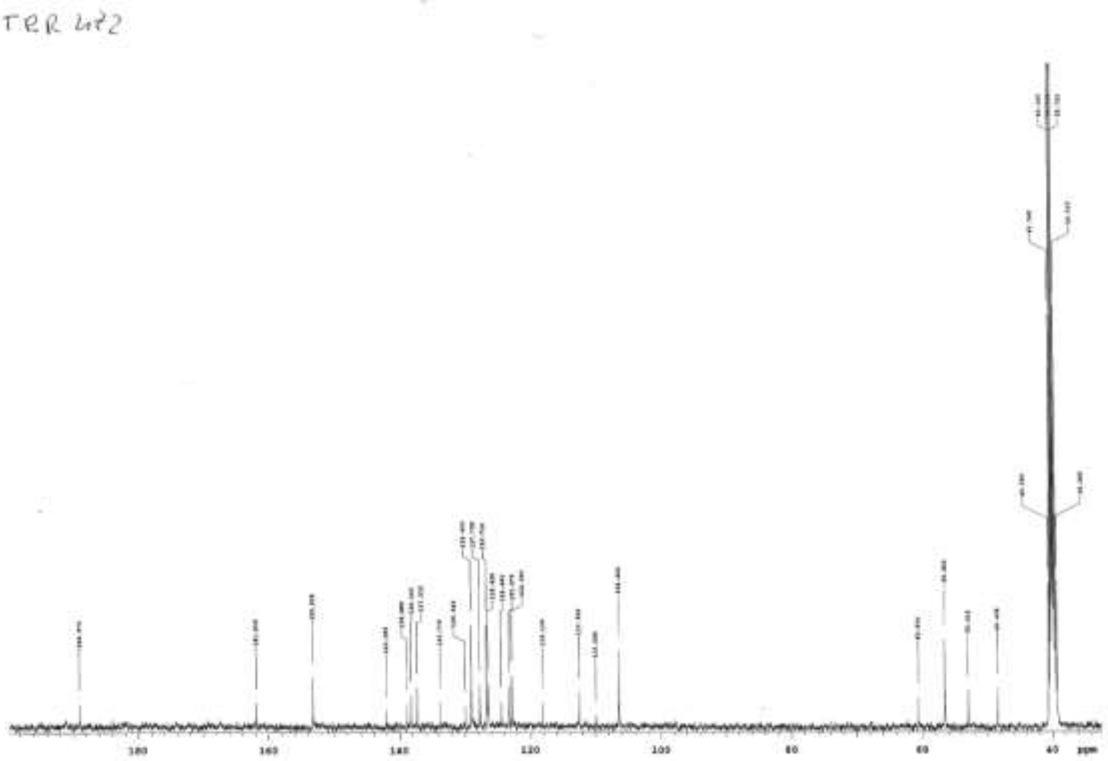

^{1}H-NMR (400 MHz, d_6-DMSO) and ^{13}C-NMR (100 MHz, d_6-DMSO) spectra of compound **9o**

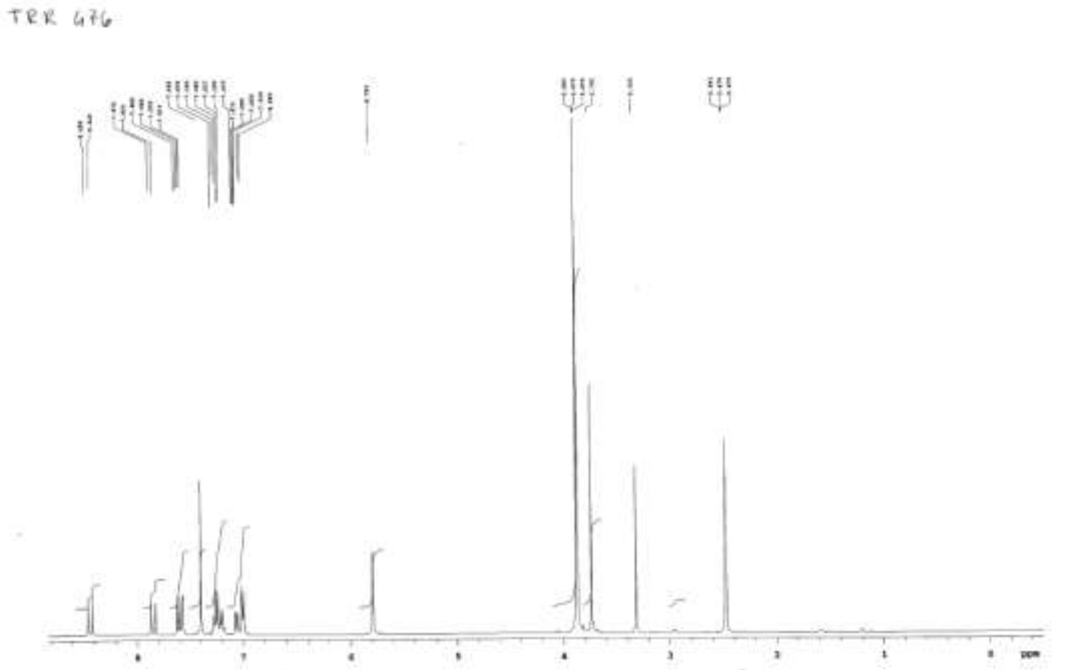

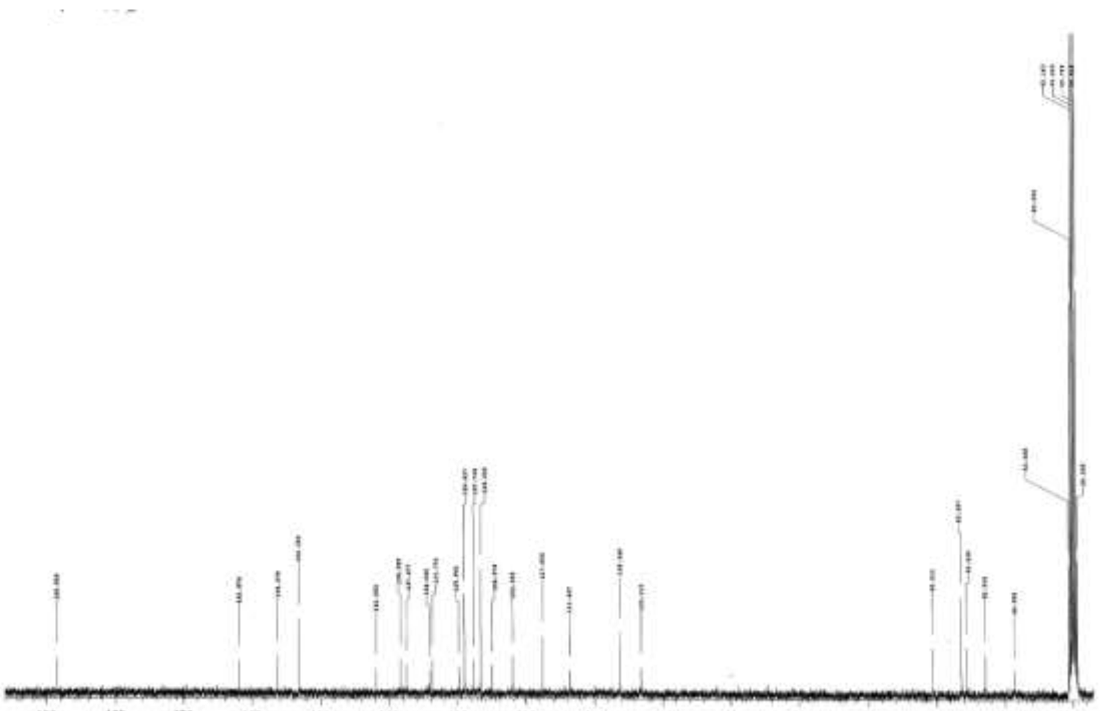

^{1}H-NMR (400 MHz, d_6-DMSO) and ^{13}C-NMR (100 MHz, d_6-DMSO) spectra of compound **9q**
